# Fast and noninvasive electronic nose for sniffing out COVID-19 based on exhaled breath-print recognition

**DOI:** 10.1038/s41746-022-00661-2

**Published:** 2022-08-16

**Authors:** Dian Kesumapramudya Nurputra, Ahmad Kusumaatmaja, Mohamad Saifudin Hakim, Shidiq Nur Hidayat, Trisna Julian, Budi Sumanto, Yodi Mahendradhata, Antonia Morita Iswari Saktiawati, Hutomo Suryo Wasisto, Kuwat Triyana

**Affiliations:** 1 Department of Child Health, Faculty of Medicine, Public Health and Nursing, Universitas Gadjah Mada, Jl. Farmako Sekip Utara, Yogyakarta, 55281 Indonesia; 2 Postgraduate Program in Clinical Medicine Science, Faculty of Medicine, Public Health and Nursing, Universitas Gadjah Mada, Jl. Farmako Sekip Utara, Yogyakarta, 55281 Indonesia; 3 Department of Physics, Faculty of Mathematics and Natural Sciences, Universitas Gadjah Mada, Sekip Utara PO Box BLS 21, Yogyakarta, 55281 Indonesia; 4 Department of Microbiology, Faculty of Medicine, Public Health and Nursing, Universitas Gadjah Mada, Jl. Farmako Sekip Utara, Yogyakarta, 55281 Indonesia; 5 PT Nanosense Instrument Indonesia, Umbulharjo, Yogyakarta, 55167 Indonesia; 6 Department of Health Policy and Management, Faculty of Medicine, Public Health and Nursing, Universitas Gadjah Mada, Yogyakarta, Indonesia; 7 Center for Tropical Medicine, Faculty of Medicine, Public Health and Nursing, Universitas Gadjah Mada, Jl. Farmako Sekip Utara, Yogyakarta, 55281 Indonesia; 8 Department of Internal Medicine, Faculty of Medicine, Public Health and Nursing, Universitas Gadjah Mada, Jl. Farmako Sekip Utara, Yogyakarta, 55281 Indonesia

**Keywords:** Predictive markers, Viral infection, Electrical and electronic engineering, Machine learning

## Abstract

The reverse transcription-quantitative polymerase chain reaction (RT-qPCR) approach has been widely used to detect the severe acute respiratory syndrome coronavirus 2 (SARS-CoV-2). However, instead of using it alone, clinicians often prefer to diagnose the coronavirus disease 2019 (COVID-19) by utilizing a combination of clinical signs and symptoms, laboratory test, imaging measurement (e.g., chest computed tomography scan), and multivariable clinical prediction models, including the electronic nose. Here, we report on the development and use of a low cost, noninvasive method to rapidly sniff out COVID-19 based on a portable electronic nose (GeNose C19) integrating an array of metal oxide semiconductor gas sensors, optimized feature extraction, and machine learning models. This approach was evaluated in profiling tests involving a total of 615 breath samples composed of 333 positive and 282 negative samples. The samples were obtained from 43 positive and 40 negative COVID-19 patients, respectively, and confirmed with RT-qPCR at two hospitals located in the Special Region of Yogyakarta, Indonesia. Four different machine learning algorithms (i.e., linear discriminant analysis, support vector machine, stacked multilayer perceptron, and deep neural network) were utilized to identify the top-performing pattern recognition methods and to obtain a high system detection accuracy (88–95%), sensitivity (86–94%), and specificity (88–95%) levels from the testing datasets. Our results suggest that GeNose C19 can be considered a highly potential breathalyzer for fast COVID-19 screening.

## Introduction

Contagious coronaviruses can cause intestinal and respiratory infections in humans and animals^1,2^. The emergence of a novel coronavirus, officially termed severe acute respiratory syndrome coronavirus 2 (SARS-CoV-2), has posed serious challenges to global health. SARS-CoV-2 infection, causing coronavirus disease 2019 (COVID-19), was found in late 2019 in Wuhan, Hubei Province, China, and subsequently spread as a causative agent for an ongoing and escalating pandemic in more than 200 countries and territories worldwide^3–5^. While the other previously found human coronaviruses (e.g., HCoV-OC43, HCoV-NL63, HCoV-229E, and HKU1) only caused mild upper respiratory diseases in immunocompetent patients, SARS-CoV-2 has been considered the third deadly pathogenic coronavirus over the past two decades after the appearances of SARS-CoV (2002–2003) in Guangdong Province, China, and the Middle East respiratory syndrome coronavirus (MERS-CoV, 2012) in Middle Eastern countries^1,6^. The COVID-19 pandemic is associated with significant fatalities, especially in the elderly and immunocompromised populations.

The reverse transcription-quantitative polymerase chain reaction (RT-qPCR) method has been routinely utilized to confirm the diagnosis of COVID-19 since the beginning of the pandemic. Thus far, this diagnostic technique detecting the ribonucleic acid (RNA) of SARS-CoV-2 has become the most widely accepted test for SARS-CoV-2 detection^7^. Various clinical samples (i.e., nasal and pharyngeal swabs, sputum, feces, blood, bronchoalveolar lavage fluid, and urine) can be employed^8^. Several companies and laboratories have developed PCR-based detection kits targeting at least two regions (genes) of the SARS-CoV-2 genome. RT-qPCR has been known for its high specificity compared to other diagnostic methods (e.g., antibody and nucleocapsid protein antigen detection assays). Nevertheless, RT-qPCR still has a few limitations (i.e., the need to be conducted by professionally trained healthcare technicians, requirement to be performed in a specialized laboratory, invasive sampling procedure, and high cost)^9^. For developed countries, conducting massive RT-qPCR examinations as a means of screening and epidemiology control is a common practice because of their abundant resources. However, for developing countries, especially with middle–low income, such features are considered to be luxurious. Nevertheless, stopping the pandemics means ensuring that any countries in the world have the capability to conduct massive and fast screening continued with selected isolation and control.

To overcome the limitation issues in using RT-qPCR as a screening tool, several clinicians and researchers have attempted to use a combination of clinical signs and symptoms, laboratory tests, imaging measurements (e.g., chest computed tomography (CT) scan), and multivariable clinical prediction models, including the electronic nose, alongside RT-qPCR for validating clinical diagnosis results^10,11^. For RT-qPCR, the detection protocol and primer sets often have to be first optimized because uncalibrated primer sets could potentially yield false-positive results^12^. Besides RT-qPCR, more than 100 kits for COVID-19 diagnosis with different working principles (e.g., isothermal amplification and lateral flow-based detection for nucleic acid targets, chest CT imaging, and immunoassays) have been produced by several companies and easily accessed on the market^13^.

Because COVID-19 primarily involves the respiratory tract, breath analysis offers an alternative as a fast and noninvasive detection approach for sniffing out this disease. It is also comfortable to perform and convenient to patients, including pediatric and elderly populations^14^. Exhaled human breath contains hundreds of volatile organic compounds (VOCs), which result from various metabolic pathways. They could be detected via gas chromatography–mass spectrometry (GC–MS) and proton transfer reaction MS^15,16^. The most abundant VOCs from exhaled human breath are acetone, methanol, ethanol, propanol, and isoprene. In addition, a few other compounds are detected in exhaled breath (e.g., benzene, acetonitrile, diallyl sulfide, allyl methyl sulfide, and diallyl disulfide)^17,18^. Generally, several VOCs with their specific compositions can be utilized as noninvasive biomarkers for various respiratory diseases, including esophageal and gastric cancers^15^, lung cancer^19^, asthma^20^, rhinovirus-induced wheezing^21^, influenza infection in swine^22^, and tuberculosis^23^. Recent evidence indicated that COVID-19 could also be diagnosed using a VOC-based breath analysis approach through near-patient GC–ion mobility spectrometry (GC–IMS)^24^. Regardless of its high accuracy, ability to precisely identify VOC types, and usefulness in pathophysiological research, the MS analysis is still time-consuming, expensive, and not user-friendly for bedside clinical practice. Moreover, for rapid COVID-19 testing of a large-scale human population, this technique alone is impractical and not feasible, especially if the target detection time should be only in a few minutes.

Therefore, an electronic nose, an artificial olfactory system consisting of an array of integrated gas sensors with different active layers and artificial intelligence (AI) to discriminate complex odors, can be opted as an alternative path to assess VOC mixtures in exhaled breaths. During its exposure to the breath, the sensors respond in a specific way to the various fractions of VOCs^25,26^. Each odor, representing a unique VOC mixture, can then result in a sensor signal pattern that is distinctive to that odor. For the exhaled air, such VOC pattern is called a breath-print. Complex VOC mixtures using pattern recognition algorithms, including machine learning, can therefore be discriminated and classified at a high throughput without identifying individual molecular components. An electronic nose is relatively inexpensive, mostly portable, fast (creating results within a few minutes), and easy to use. Considering patient perspectives in the case of COVID-19, exhaled breath analysis using an electronic nose is attractive because it is noninvasive, rapid, safe, and simple to operate. Previously, the electronic nose was implemented in clinical settings to diagnose and monitor respiratory and urologic diseases (e.g., ventilator-associated pneumonia, tuberculosis, and kidney failure), and recently, it has been used as a pre-operative screening for COVID-19^27–30^. VOC profiles are expected to change following the continuous progression of COVID-19 within patients’ respiratory system. Their alteration is complex, which is associated with the SARS-CoV-2 and its unique interaction with the host.

Depending on their working principles and materials, several thin-film and micro-/nanoscale gas sensors can be employed as the main detecting components in the electronic nose, which include gravimetric sensors (e.g., surface acoustic wave resonators, resonant micro-/nanocantilevers, and quartz crystal microbalances)^31–40^, chemoresistive sensors (e.g., metal oxide semiconductor, polymer, two-dimensional material, and carbon-based sensors)^41–46^, colorimetric sensors (e.g., printable pastes and optical dye-functionalized washable threads)^47,48^, and optical sensors (e.g., visible and infrared micro-/nano-light-emitting diodes (micro-/nanoLEDs), plasmonic lasers, and femtosecond lasers)^49–56^. Among them, chemoresistive metal oxide semiconductor gas sensors have been favorable, especially for VOC detection, due to their excellent characteristics (i.e., low cost, short response time, simple measurement setup, high durability, and long lifetime)^57^. The drawback of such sensors concerning the moderate selectivity can be overcome when different types of active materials are used in a simultaneous fashion. Their unique signals can then be identified and combined in AI-based data post-processing. Hence, the electronic nose comprising an array of chemoresistive metal oxide semiconductor sensors has been used in many environmental monitoring fields^41,45^.

The evidence that electronic nose technologies can be potentially employed to detect and analyze the breath pattern signature of COVID-19 resulting from the VOC-based target biomarkers has been reviewed in another study^58^. An electronic nose comprising commercial metal oxide semiconductor-based sensors (i.e., MQ-135 and MQ-2) capable of detecting gas concentrations in parts per million (ppm) of carbon monoxide (CO), acetone, and alcohol, were used in simulating the possible detection of COVID-19. The proposed technology had been patented by the National Aeronautics and Space Administration in the United States as a portable unit for metabolic analysis^45,59^. As the infectious disease (COVID-19) improved, diagnostic electronic nose systems were suggested to include a diversity of metal oxide semiconductor-based gas and electrochemical sensors to enable accurate monitoring of exhaled breath biomarkers of disease and modifications associated with the disease pathogenesis^45,58^.

**Fig. 1:**
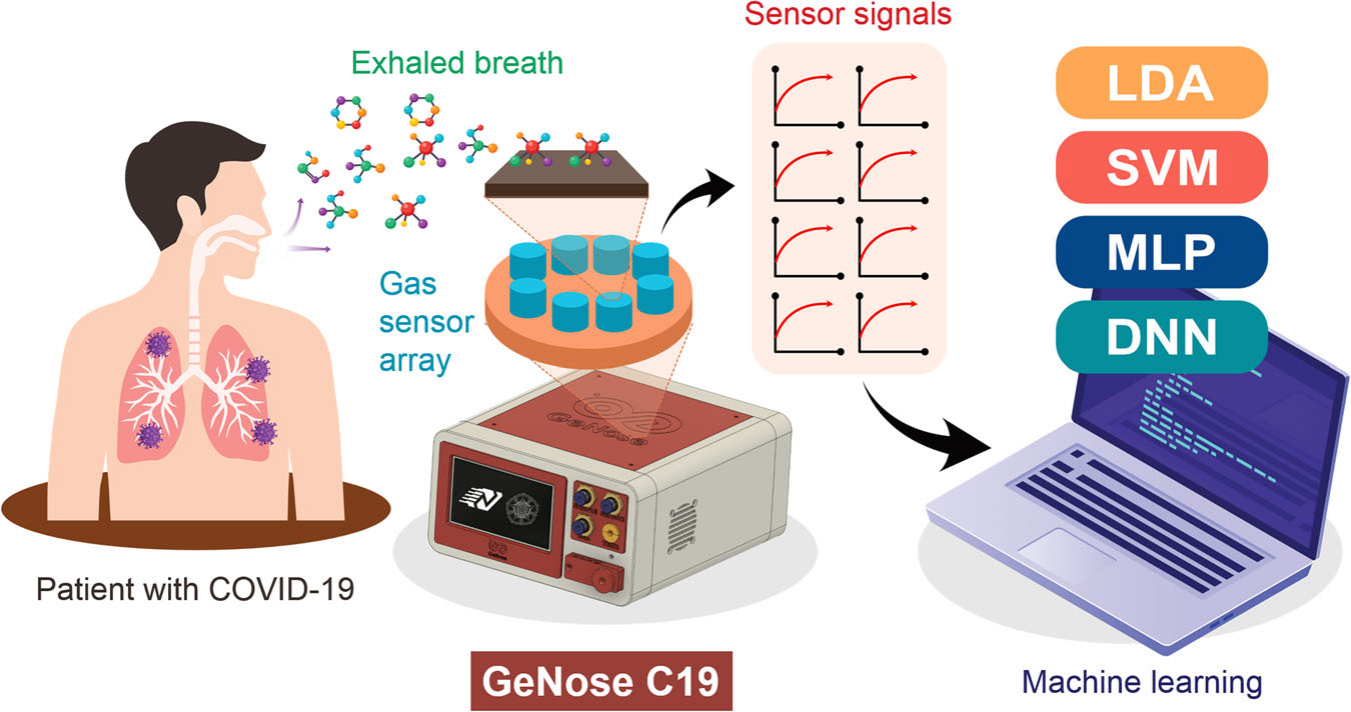
Illustration of the fast and noninvasive COVID-19 detection utilizing portable electronic nose (GeNose C19) integrated with artificial intelligence (AI). Four different machine learning algorithms (i.e., linear discriminant analysis (LDA), support vector machine (SVM), stacked multilayer perceptron (MLP), and deep neural network (DNN)) were employed to differentiate and classify the exhaled breath patterns of the patients, which were measured by 10 different metal oxide semiconductor gas sensors.

To the best of our knowledge, so far, no published study has investigated the potential of electronic noses in detecting COVID-19 patients at the bedside clinical setting. Therefore, in this work, we developed a portable breathalyzer, so-called GeNose C19, by integrating an array of metal oxide semiconductor gas sensors, machine learning analysis, and breath sampling setup (see Fig. 1). The custom-built system was employed in profiling clinical tests involving two different subject groups (i.e., RT-qPCR-confirmed positive and negative COVID-19 patients) in two hospitals located in Indonesia to investigate its potential for differentiating the exhaled breath patterns of both groups. Four different machine learning algorithms were examined to determine the highest possible accuracy of the developed device.

## Results

### Integrated electronic nose for COVID-19 detection (GeNose C19)

**Fig. 2:**
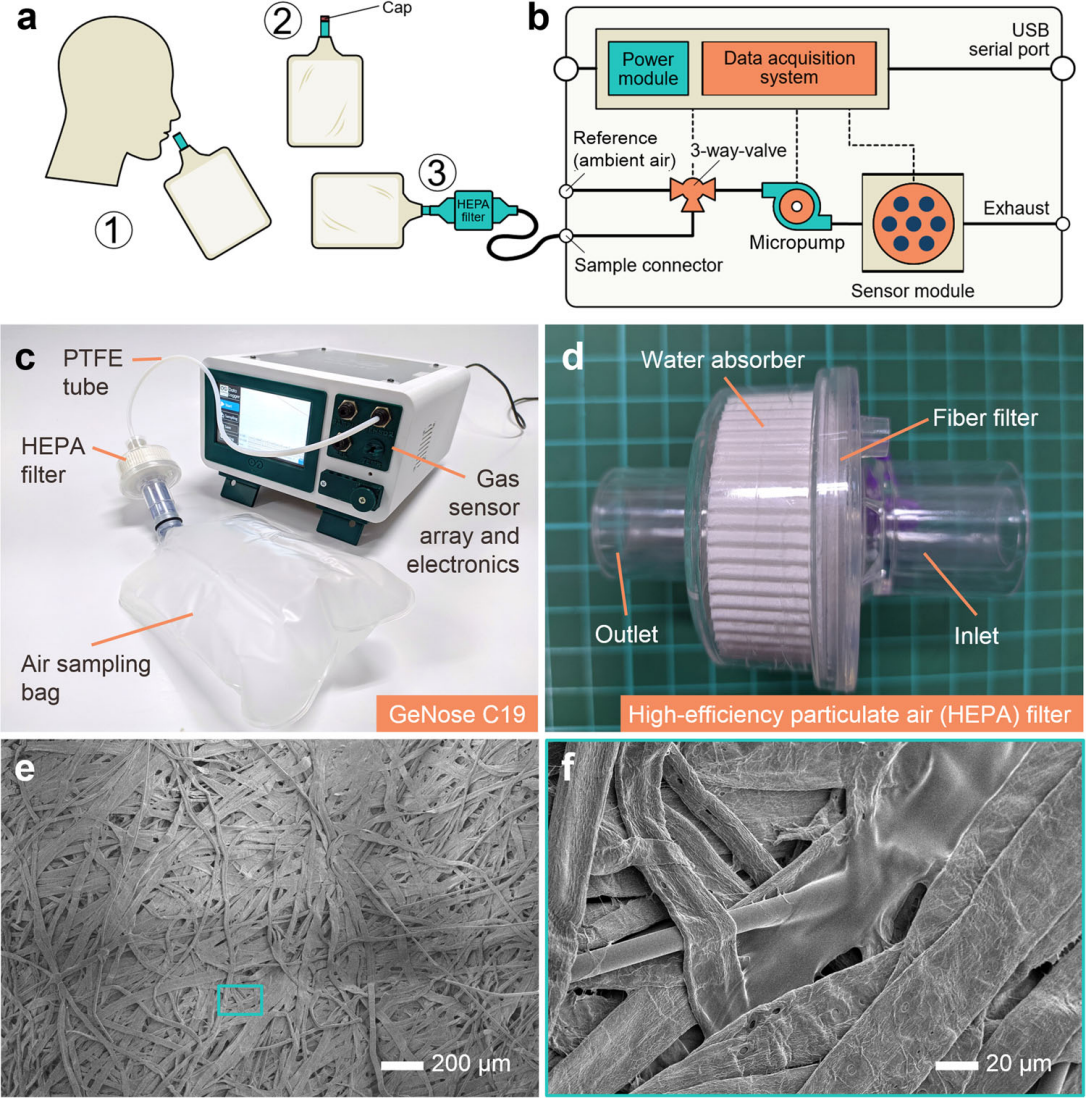
Integrated GeNose C19 system and its components. **a** Scheme of the off-line breath sampling pipeline for GeNose C19: (1) air inhaled through the nose and subsequently exhaled through the mouth to a sampling bag, (2) sealing or closing of the sampling bag cap to avoid the collected air leakage, and (3) direct plugging of the sampling bag into the electronic nose inlet (sample connector). **b** Diagram and **c** photograph of GeNose C19 integrated with a high-efficiency particulate air (HEPA) filter and an air sampling bag through a flexible medical-grade polytetrafluoroethylene (PTFE) tube with an outer diameter of 4 mm. The electronic nose consists of several main electronic and mechanical parts (i.e., power module, data acquisition system, 3-way-valve, micropump, and sensor module inside the sealed gas chamber). The sensor module comprises 10 different sensing devices arranged as an array. **d** HEPA filter for filtering out the particulate matters and trapping the SARS-CoV-2 available from the exhaled breath of a patient confirmed with positive COVID-19. **e**, **f** Scanning electron micrographs of the fiber filter used in the GeNose C19-filtering system.

The electronic and mechanical components integrated into an electronic nose for sniffing out COVID-19 (GeNose C19) were grouped into two main parts (i.e., sensing and breath sampling units), as depicted in Fig. 2a, b. The former consists of a chemoresistive sensor array sealed in a miniaturized chamber, micropump, three-way solenoid valve, power supply, and data acquisition system. All of them were placed in a three-dimensional (3D)-printed housing. Meanwhile, the latter comprises a high-efficiency particulate air (HEPA) filter and a disposable air sampling bag made of medical-grade polyvinyl chloride. Both units were designed separately and then connected by utilizing a flexible medical-grade polytetrafluoroethylene (PTFE) tube with an outer diameter of 4 mm to enable airflow during gas sensing (see Fig. 2c). Here, the HEPA filter was attached between the reservoir bag and GeNose C19 inlet to filter out virus-containing droplets. Moreover, it possessed a water absorber element to eliminate water molecules, which are one of the most interfering factors for gas sensors (Fig. 2d). Hence, only the target VOCs will enter the sensing chamber because the viruses are expected to be trapped by the filter containing fibrous mats (see Fig. 2e, f) and are not allowed to contaminate the whole air trajectory in the GeNose C19 machine.

To ensure human safety during the breath exposure test and obtain reliable results, the breath sampling procedure was carefully set and controlled (Fig. 2a). First, the patients were asked to store their end-tidal breath during the third exhalation into 1 L sampling bags (see Fig. 2a(1)). Here, the first two breaths were not taken to minimize contamination from the dead space air (i.e., ventilated air that does not participate in the gas exchange) and mouth-released odor, which are commonly found in the mixed expiratory breath^60^. From another breath analysis report, different sampling procedures (i.e., mixed expiratory and end-tidal breath sampling methods) were discovered to result in varied VOC ratios^61^. In case the blood-borne volatile substances will be assigned as biomarkers of disease, such as in COVID-19 detection, the end-tidal breath sampling technique is, however, appropriate to be used^60^. The ratio values between the difference in the expiratory and inspiratory concentrations and the alveolar concentration were normally calculated to estimate the content alteration of inhaled and exhaled substances, where blood-borne volatile substances with clearly endogenous (e.g., CO_2_, isoprene, and acetone) and exogenous origins (e.g., 2-propanol and 2-butanone) obtained high (>1.5) and low (<1) ratios, respectively^61,62^. After the third exhaled breath had been inserted into the sampling bag, its valve had to be quickly closed to avoid any air leakages (see Fig. 2a(2)). Lastly, the upper end of the reservoir tube was connected to the HEPA filter. Hence, the valve could subsequently be reopened, allowing the airflow to occur from the bag to the GeNose C19 machine (Fig. 2a(3)). Such a similar breath sampling concept has already been employed in various off-line breathomics pipelines^60^.

Depending on the used filter, having multiple stacks of combined micro-/nanofibers with diameters in the range of <1–50 µm (see Fig. 2d–f) can ensure the successful filtration of bioaerosols carrying RNA copies of SARS‐CoV‐2. Different mechanisms are responsible for the final sizes of the emitted virus-containing particles, which were found to be ranging from <1 to >100 μm when humans performed different actions (i.e., breathing, talking, sneezing, and coughing)^63^. For normal breathing, particles with diameters of <0.8–2.0 μm were mostly observed^63,64^. In other clinical investigations, a similar high-grade HEPA filter was placed between the bag valve and patient mask during preoxygenation in the intubation procedure for patients with COVID-19^65^. This filtering technique was also used to safely deliver aerosolized medications to COVID-19 patients, avoiding the aggravation of the novel coronavirus spreading^66^. Despite the adequate proofs of the HEPA filter effectiveness in trapping the aerosolized virus-containing particles from other reports^65,66^, we conducted additional investigations for the air trajectory part (pneumatic PTFE tube) after GeNose C19 was used to measure the exhaled breath of a confirmed positive COVID-19 patient. Our examinations revealed that the taken sample of the inner tube part was confirmed negative in the RT-qPCR test, which indicates the successful removal of the coronaviruses from the airways in the fiber filter. Thus, they did not contaminate the other components inside the sensing unit. In other words, the exhaust air produced by GeNose C19 can be safely released to the ambient environment and will not further transmit airborne viruses.

In the sensing unit, 10 different gas sensors based on metal oxide semiconductors (S1–S10) were integrated with internal heaters as a detection module, where each of them had unique cross-sensitivity characteristics to several VOCs. As a result, a specific signal pattern that represents the exhaled breath from either RT-qPCR-confirmed positive or negative COVID-19 patients was obtained. Because each sensor requires varied power consumptions between 280 and 835 mW, a total power of ~6 W was supplied to operate the whole sensor module. Moreover, GeNose C19 was equipped with an environmental sensor to monitor possible interferences from water molecules (humidity) and temperature changes inside the sensing chamber. For enabling and controlling the alternating flows of reference (ambient) air and exhaled breath to the chamber, a micropump and a three-way solenoid valve were integrated into the GeNose C19 machine, respectively. While the micropump could create an air flowrate of (1 ± 0.2) L/min, the solenoid valve could be configured into three modes (i.e., delay, sampling, and purging phases). First, during the delay phase of 10 s, the ambient air was drawn by the micropump into the test chamber and subsequently characterized by the sensor array as the baseline voltages. Second, a VOC-containing breath sample was piped from the reservoir bag into the test chamber in the sampling phase. It then reacted with the sensors within 40 s, yielding various response signals. Third (purging phase), the ambient air was again re-inserted into the chamber for 120 s to remove the remaining VOCs. The produced exhaust air was piped out to the ambient via the outlet located at the rear of the GeNose C19 machine. The microcontroller and 16-bit external analog-to-digital converter were employed in the data acquisition system module to control the data exchange within electronics and convert the analog sensor responses (in voltage) to digital values, respectively. Hence, the digital data could finally be transferred to a homebuilt data logging software in the personal computer through a Bluetooth or universal serial bus connection for further analysis.

Because the sensors are sensitive to variations in atmospheric conditions, the initial placement of the GeNose C19 system can vary the output sensing results. In this case, indoor environments with well-ventilated indoor spaces or outdoor areas with poor air circulation (i.e., rooms with strong odor backgrounds originating from different sources, such as fragrances, alcohol-based hand sanitizers, cleansers, car exhausts, wall paints, certain foods, and beverages) will affect the performance of GeNose C19, resulting in poor or peculiar signals. Moreover, too high changes in the room temperature and humidity can affect the measurement results. Thus, the GeNose C19 machine has to be carefully preconditioned before its use, as described in Methods (GeNose C19 preconditioning). The preconditioning protocols include environment preparation and modification, machine placement and pre-operation, and fasting requirement for patients before the breath examination.

### Sensor characteristics to exhaled breaths

The sensor baseline values were first measured where no breath was piped into the test chamber (i.e., the sensor signals corresponding to the ambient condition). They would then be used as subtrahends for the output response signals to the VOC-containing breath. After the gas molecules interacted with the active layer surfaces of the chemoresistive sensors, their output voltages increased due to the reduced electrical resistances. The sensing measurements were simultaneously conducted for all the sensors within 40 s to reach their saturation stages. Commonly, for metal oxide semiconductor gas sensors, their conductivity will change in the presence of target gases due to a redox reaction between the active material (e.g., *n*-type tin oxide (SnO_2_)) and gas molecules. The detailed sensing mechanism toward VOCs based on an equilibrium shift of the surface chemisorbed oxygen reaction has been described in other studies^41,67–69^. Here, the depletion regions at material surfaces were controlled by target VOCs, leading to a change in the movement of free charge carriers from the metal oxide semiconductor to the oxygens or vice versa (i.e., free electrons in the case of the *n*-type SnO_2_ active layers).

**Fig. 3:**
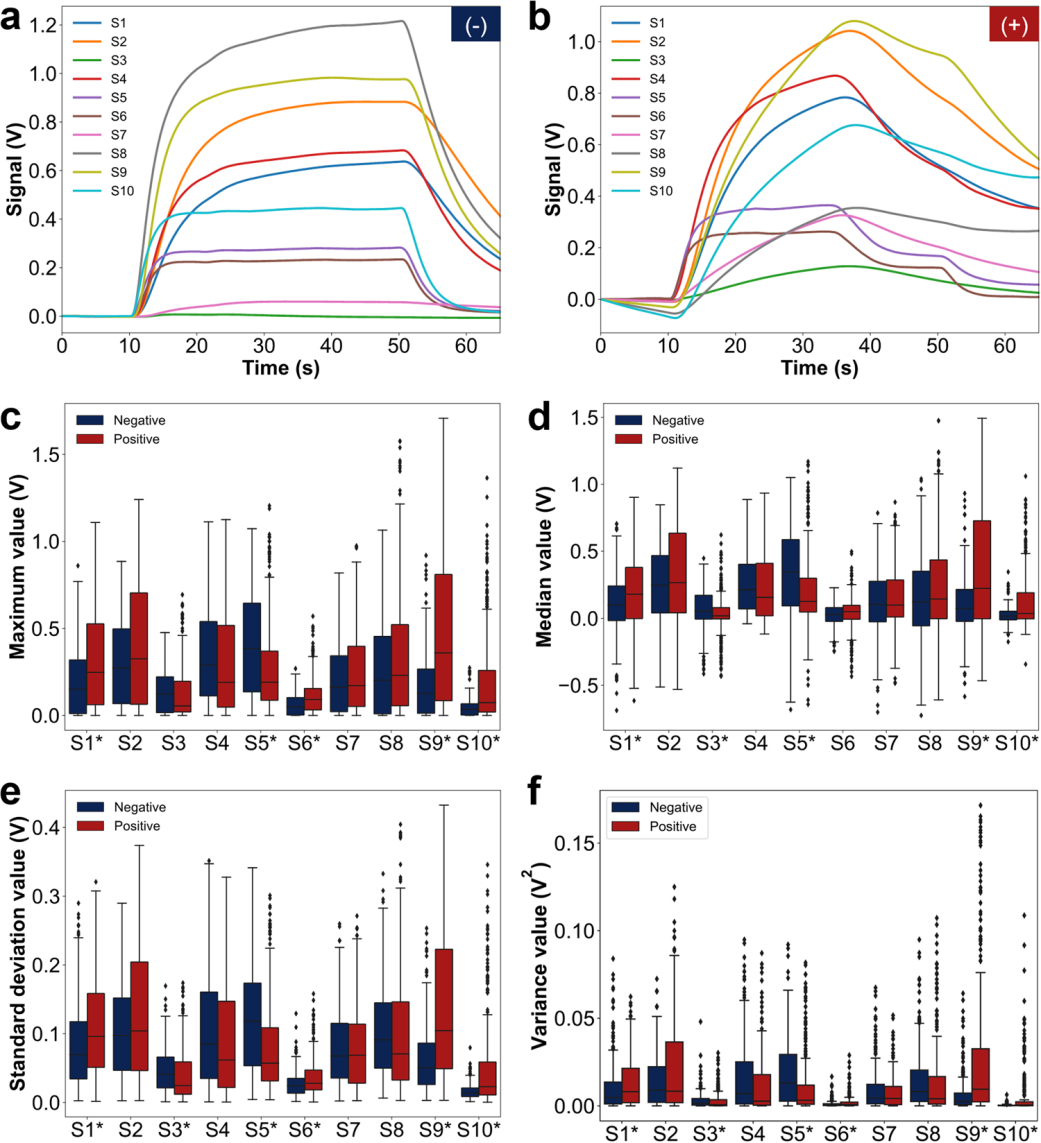
GeNose 19 sensor responses and their extracted features from the exhaled breaths of RT-qPCR-confirmed negative and positive COVID patients. Typical sensing responses were obtained from 10 different conductometric gas sensors (S1–S10), which are integrated into a portable GeNose C19 system for the exhaled breaths of RT-qPCR-confirmed **a** negative and **b** positive COVID-19 patients. Boxplots of the distribution for negative and positive COVID-19 samples based on feature extraction: **c** maximum, **d** median, **e** standard deviation, and **f** variance values.

Initially, when the activated sensor was exposed to ambient air, oxygen molecules were adsorbed on the metal oxide semiconductor surfaces. The free electrons inside the sensing material were then attracted and attached to those oxygens. As a result, the measured electrical resistance increased, and the sensor output voltage dropped into a lower value. This condition can be seen when the GeNose C19 sensors were preheated for at least 20–50 min in ambient air (see Supplementary Fig. 1). At this elevated temperature, sensor output voltage reductions ranging from 0.24 to 2.74 V were yielded depending on the initial measured baseline values and type of functional materials. Afterward, in the presence of reducing gases or VOCs (e.g., propane and formaldehyde (HCHO)), the VOC molecules reacted with the adsorbed oxygen ions, resulting in a low number of oxygen on the semiconductor surfaces. Consequently, the previously trapped electrons were released back to the metal oxide semiconductor, leading to a high free electron concentration and low sensor electrical resistance. For the sensor output voltages, a rise in their values was also observed, while the sensors interacted with the VOCs contained in the exhaled breaths of the RT-qPCR-confirmed negative and positive COVID-19 patients (see Fig. 3a, b, respectively). After ~10–40 s sensing, the sensor signals became stable, indicating material surface saturation. A high VOC concentration injected into the chamber led to a low concentration of the adsorbed oxygen on the semiconductor surface, which then further reduced the sensor resistance. Such a phenomenon was employed to realize a real-time monitoring of VOCs in the air^70^. Once exposure of the sensor to the exhaled breath had ended (i.e., the sensing chamber inside the GeNose C19 machine was flushed with ambient air), the free electrons inside the metal oxide semiconductor returned, attaching to the adsorbed oxygens on surfaces. This was followed by the recovery of the response signals to their initial values (baselines). Here, the recovery duration is longer than the response time, which can be attributed to the required time to break the existing bonding between the remaining VOCs and oxygen molecules on the sensing surfaces.

Figure 3a, b shows the typical responses of the gas sensor array in GeNose C19 to the breaths of negative and positive COVID-19 patients, respectively. From these typical sensing signals, it is not easy to distinguish them directly and visually. Therefore, we extracted the features of each sensor based on the combination of the maximum, median, standard deviation, and variance values, which resulted in 40 features for each patient data. Figure 3c–f depict the distribution boxplots for the positive and negative label classes. In this study, no error bar was used. For each boxplot, the line shows the distribution of data with values ranging from (Q1–1.5 × IQR) to (Q3–1.5 × IQR), where IQR = Q3–Q1. Here, Q1, Q3, and IQR are the first quartile, third quartile, and interquartile range, respectively. A non-parametric statistical test (i.e., Kruskal–Wallis test) was used to determine the significance of the difference between the negative and positive patient group data. This test is commonly used as an alternative to the one-way ANOVA test, where the assumption of normality of the data is not met. Sensors showing significant group population differences are indicated by an asterisk (*) symbol in the boxplot label. More detailed feature values are listed in Supplementary Table 1. The test results demonstrated that the positive and negative groups could be significantly distinguished (*p* value < 0.05) by the maximum, median, standard deviation, and variance values of S1, S5, S6, S9, and S10.

### GeNose C19 performance in COVID-19 profiling tests

**Fig. 4:**
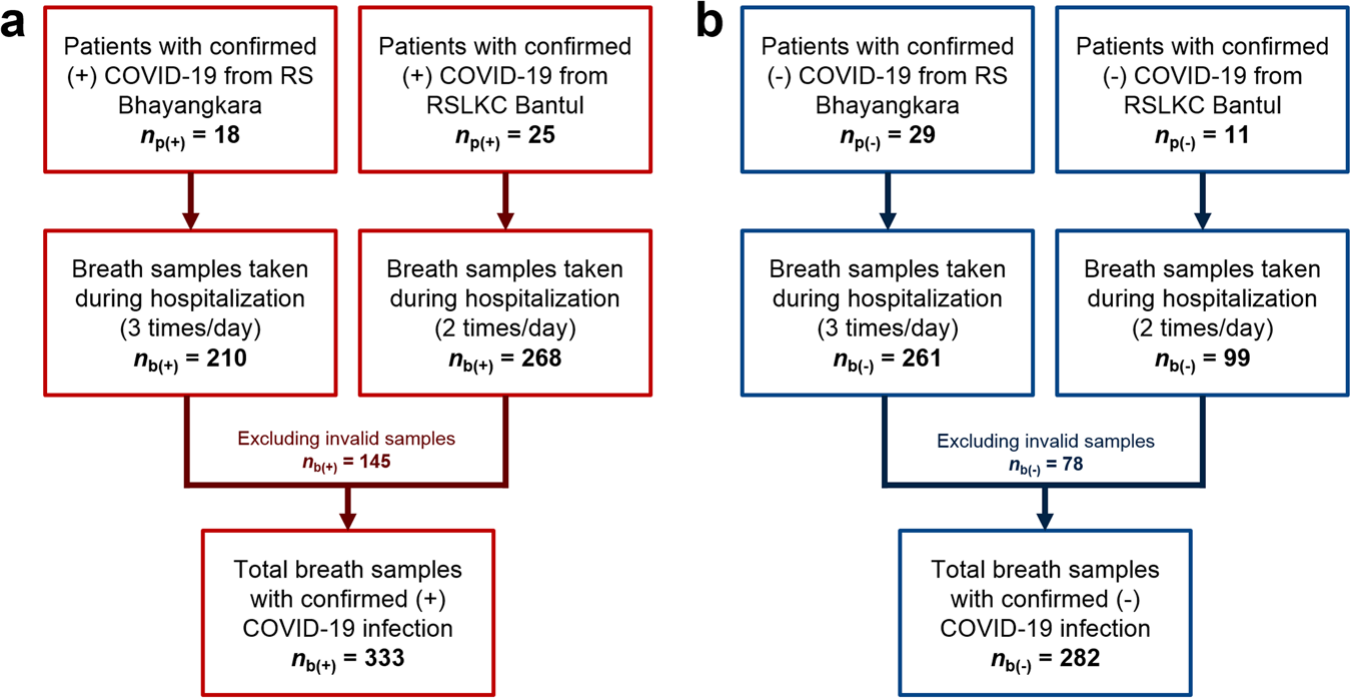
Exhaled breath collection procedure performed in the profiling test. Breath sampling procedure comprising patients with RT-qPCR-confirmed **a** positive (*n*_p(+)_) and **b** negative (*n*_p(−)_) COVID-19 infection in two hospitals (i.e., Bhayangkara General Hospital in Sleman District (RS Bhayangkara) and Bambanglipuro COVID-19 Special Field Hospital in Bantul District (RSLKC Bantul)). Both are located in the Special Region of Yogyakarta, Indonesia. The total breath samples were obtained by excluding the invalid ones measured by GeNose C19, in which the total confirmed positive and negative COVID-19 samples were *n*_b(+)_ = 333 and *n*_b(−)_ = 282, respectively.

GeNose C19 was employed in COVID-19 profiling tests at two hospitals (i.e., Bhayangkara General Hospital in Sleman District (RS Bhayangkara) and Bambanglipuro COVID-19 Special Field Hospital in Bantul District (RSLKC Bantul)) to investigate its potency and functionality for COVID-19 detection. Eighty-three subjects were recruited, consisting of 43 and 40 subjects confirmed as positive COVID-19 (case group) and negative COVID-19 (control group), respectively (see Fig. 4). Two subjects from the case group were excluded due to the deterioration of their clinical conditions, and they were referred to high-level healthcare facilities. More details on the profiling test procedure are presented in Methods.

In terms of the clinical characteristics of the tested patients, Table 1 shows that 79–80% of the COVID-19 positive patients were asymptomatic. Symptomatic patients here refer to patients admitted and manifesting COVID-19 suggestive symptoms, including cough, sneezing, fever, and history of contact with other positive patients. However, they were classified into either positive or negative COVID-19 group based on the results from two-time RT-qPCR examinations. In the early phase of the COVID-19 pandemic in Indonesia (June–September 2020), the Health Ministry issued a policy stating that all persons identified to have COVID-19 infection based on the contact screening and RT-qPCR examination, had to be isolated in the hospital. Thus, some of the recruited patients were asymptomatic, whereas others exhibited mild symptoms of COVID-19 (e.g., cough, sneezing, anosmia, and fever) at the time-of-admission. The estimated mean length of stay was (9.0 ± 1.6) days. Breath samples were obtained by excluding the invalid ones measured by GeNose C19, in which the total confirmed positive and negative COVID-19 breath samples were *n*_b(+)_ = 333 and *n*_b(−)_ = 282, respectively. Here, although most patients were asymptomatic, the difference in the VOC patterns could be distinguished. Most participants are aged 18–44 years with a mean age of 32.5 years without pre-existing comorbidities. The existing comorbidities were evenly distributed within both groups in Table 1 (i.e., cardiovascular problems, allergy and atopic conditions, and endocrine metabolic problems). None of them had respiratory diseases.

**Table 1:** Clinical characteristics of the tested patients, including age, sex, comorbid condition, symptoms, and breath sample type.

Characteristics	RT-qPCR-confirmed positive COVID-19 (*n*_p(+)_ = 43)	RT-qPCR-confirmed negative COVID-19 (*n*_p(−)_ = 40)	Total number	*p* value
Age distribution (years old) Mean age: 36 ± 4				0.239
0–17	2	0	2	
18–44	27	22	49	
45–64	13	18	31	
65–74	1	0	1	
Sex distribution				0.000
Male	30	6	36	
Female	13	34	47	
Comorbidities				0.554
Cardiovascular problems	4	3	7	
Allergy and atopic conditions	0	1	1	
Endocrine metabolic problems	1	0	1	
Respiratory problems	0	0	0	
No pre-existing comorbid	38	36	74	
Symptoms				0.667
Patients with symptoms	9	6	15	
Asymptomatic patients	34	34	68	
Breath samples				
Positive COVID-19 detected	318	12	330	
Negative COVID-19 detected	15	270	285	

All the positive and negative COVID-19 patients have been tested and confirmed using reverse transcription-quantitative polymerase chain reaction (RT-qPCR). *P* value is measured using a non-parametric statistical test (i.e., Kruskal–Wallis test), where *p* value < 005 is considered to be statistically significant.

During machine learning and AI optimization, 70% of the breath samples were used as training materials for the AI-based software validation. The remaining 30% of samples were employed for accuracy testing using the first 70%. Later, all breath samples (100%) were processed to measure the sensitivity and specificity levels of all machine learning algorithms (i.e., support vector machine (SVM), stacked multilayer perceptron (MLP), deep neural network (DNN), and linear discriminant analysis (LDA)). The ones that were previously employed in the training were re-tested using all four AI models. Table 2 lists the most important parameters (i.e., sensitivity, specificity, and area under the curve (AUC)) of GeNose C19 obtained from all datasets processed with the DNN model, which is considered the most stable and optimum machine learning algorithm among others. The sensitivity and specificity levels were 95.5% (95% CI: 92.7–97.3%) and 95.7% (95% CI: 92.7–97.5%), respectively. Meanwhile, the AUC was 95.6% (95% CI: 93.7–97.1%). The calculated values show the reliability of the DNN algorithm in predicting the training and testing data of the breath samples.

**Table 2:** Key parameters of GeNose C19 obtained from the optimum machine learning algorithm (DNN) of all breath samples (training and testing datasets).

2 × 2 Tables		RT-qPCR		Total	Sensitivity (95% CI)	Specificity (95% CI)	AUC (95% CI)
		(Positive)	(Negative)				
GeNose C19	(Positive)	318	12	330	95.5% (92.7%–97.3%)	95.7% (92.7%–97.5%)	95.6% (93.7%–97.1%)
	(Negative)	15	270	285			
Total		333	282	615			

These include sensitivity, specificity, and area under the curve (AUC).

**Fig. 5:**
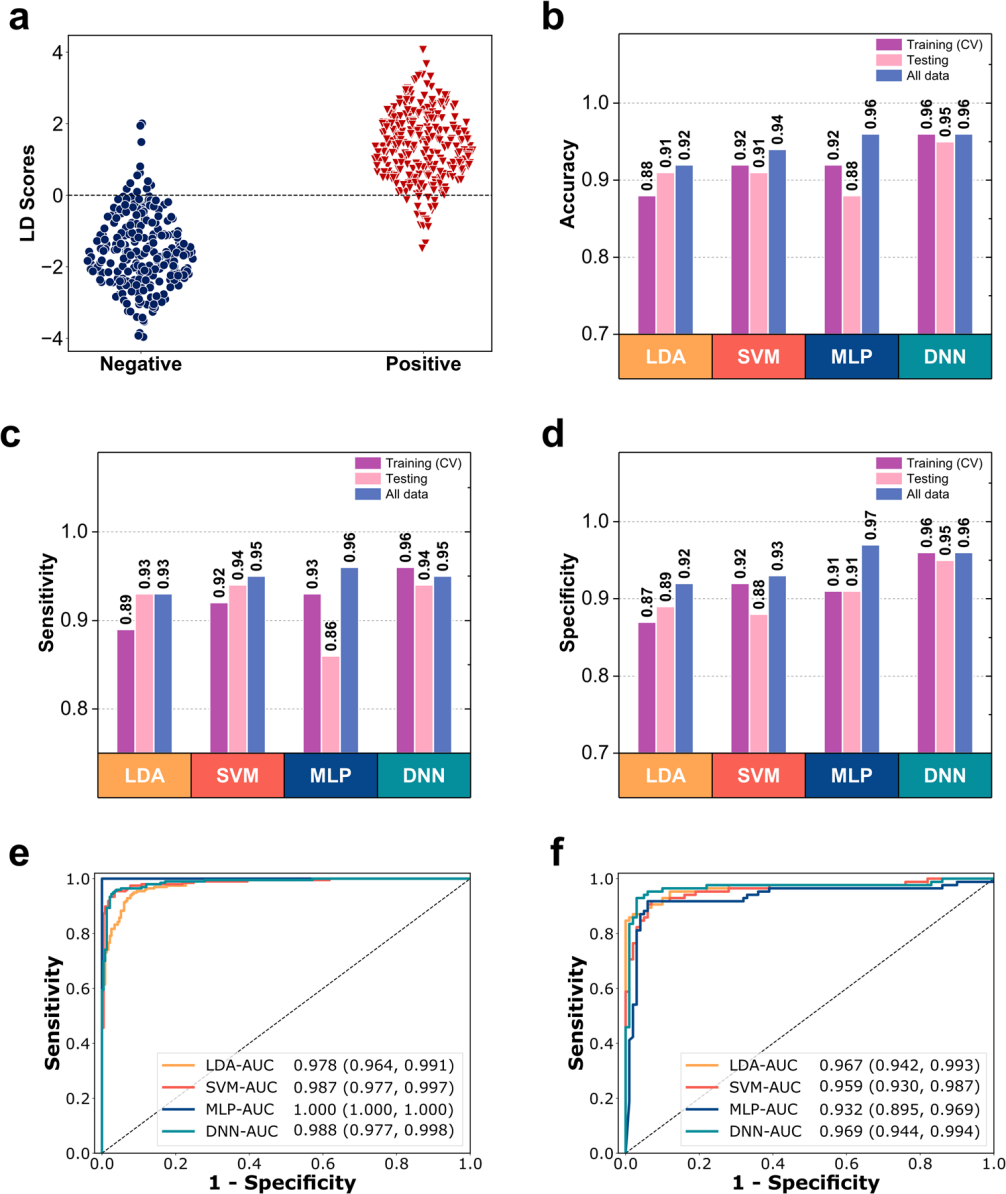
Measured breath data analysis using machine learning. **a** Classification of two different exhaled breath samples (i.e., positive and negative COVID-19 samples) using the LDA model. **b** Overall accuracy (micro-averaged F1-score), **c** and **d** are sensitivity and specificity, respectively, of the training (10-fold cross-validation), testing, and all datasets obtained by four different machine learning algorithms (LDA, SVM, MLP, and DNN). **e** and **f** are the receiver operating characteristic (ROC) curves of the training and testing data, respectively, obtained by four different machine learning algorithms (LDA, SVM, MLP, and DNN). AUC and confidence intervals are also shown.

Before selecting the four machine learning models (i.e., LDA, SVM, MLP, and DNN) to be used in GeNose C19, we also investigated other learning algorithms (e.g., gradient boosting, decision tree, and regression algorithms). The results from the gradient boosting and decision tree models were considered to have a very high risk of overfitting, resulting in the high accuracy in the internal validation but low performance in the external validation. Meanwhile, the regression method did not fit in the classification model of diagnostics proposed in this study. Particularly, Fig. 5 has been presented as such to show the performance and reliability of different machine learning algorithms when they read the testing data compared to the training data. The smaller the changes between the training and testing data, the more reliable and consistent the AI protocols. Here, again, the DNN algorithm showed the highest similarity in terms of sensitivity, specificity, and accuracy between the training and testing data, indicating that it has the optimum performance among other algorithms.

From Supplementary Fig. 5, the principal component analysis plots of the first three principal components naturally did not show any separation between the positive and negative groups. Thus, the focus of the next analysis was to use supervised machine learning models of LDA, SVM, MLP, and DNN. The classification of two different exhaled breath datasets (positive and negative) was first performed using LDA to simplify their high-dimensional data complexity while keeping patterns and trends. We transformed the obtained data into fewer dimensions acting as feature summaries. LDA is a supervised learning method that can also reduce dataset dimensions. LDA has focused more on the characteristics of different classes to discriminate them, in which each class can then be optimized^71^. Figure 5a shows the plot of the optimized LDA analysis for all the exhaled breath data. The results indicate that the top two classes that can be well-distinguished with a small portion are still overlapping.

All datasets obtained during the sensing phase in the COVID-19 profiling test were utilized as inputs for all four classification models. To control the learning process, the hyperparameters were optimized for the SVM, MLP, and DNN models. Furthermore, the grid-search optimization algorithm (i.e., linear kernel function and radial basis function) was applied to tune the SVM parameters. In the optimized results of the dataset preprocessing by StandardScaler, the cost value is 10, and the kernel function is linear. We also applied a genetic algorithm for tuning the hyperparameters of MLP and DNN. In the case of the MLP model, a tree-based pipeline optimization tool (TPOT) was implemented, which is basically an automated machine learning library. This TPOT library allowed the selection of a stacked MLP model with MinMaxScaler for preprocessing the dataset. Finally, to optimize the DNN model, we tuned the epochs, number of batches, hidden layer, neuron, dropout value, optimizer function, and activation function. A sigmoid function was used for the last activation function with a binary cross-entropy loss. The applied procedure allowed us to employ 500 epochs, a batch size of 5, the first hidden layer with 500 neurons and the “relu” activation function, the first 0.1 dropout layer, the second hidden layer with 250 neurons and the “relu” activation function, the second 0.1 dropout layer, and the “adam” optimizer function. The validation size of 0.2 and five early stopping procedures were used in the DNN training procedure to avoid overfitting. In the hyperparameter tuning procedure, data training and 10-fold cross-validation were applied. After obtaining the optimum parameters, the classification model was trained using training data and subsequently evaluated by testing data.

The performance of each machine learning model was represented by three types of metrics (i.e., sensitivity, specificity, and overall accuracy). Sensitivity and specificity are the correctly classified proportions of actual positives and negatives, respectively. Meanwhile, accuracy is the correctly classified proportion of combined actual positives and negatives. Figure 5b–d shows the accuracy, sensitivity, and specificity of all datasets measured by GeNose C19 during the profiling tests. The internal validation of the machine learning model was conducted by applying a repeated 10-fold cross-validation technique. This method ensures that 70% of the training data are used for internal validation purposes in each iteration and the other 30% of the data are used for testing purposes at each run. The DNN model had the most stable performance among the others, which was indicated by the high and similar accuracy values between the training and testing data.

In addition to the two performance parameters (sensitivity and specificity), we also investigated the receiver operating characteristic (ROC) curves with their resulting AUC values of the training (Fig. 5e) and testing data (Fig. 5f) for all the machine learning models. Among the machine learning models, the DNN yielded the most stable performance indicated by the high and similar AUC values between the training and testing data of 98.76% and 96.87%, respectively. In particular, even though 615 breath samples were collected repeatedly from 83 patients, we provided the clinical characteristics of the patients on the basis of the first examination at the time-of-admission only. The data were collected and analyzed as such to gain insights into the pattern profile difference between the positive and negative COVID-19 groups, explore the VOC pattern change during hospitalization and disease progression, and collect the materials for AI training and performance testing.

Furthermore, we performed additional AI analysis on the VOC pattern on the basis of individual subjects (not on the basis of breath samples). Despite a different approach, similar results and performance of machine learning models are shown in the analysis using a single breath sample from each subject (83 samples in total), as depicted in Supplementary Fig. 6. Here, the DNN also showed the most stable performance, indicated by the similar AUC values between the training and testing data of 99.9% and 96.9%, respectively. Supplementary Table 2 exhibits a pairwise difference between the classification models based on Cohen’s d equation. The differentiation between the algorithm and classification models is shown in terms of accuracy, sensitivity, and specificity. A large difference in the performance was demonstrated by the DNN as compared to the other models (LDA, SVM, and MLP).

## Discussion

For the GeNose C19 sensor array, the sensitivity of each sensor during exposure to varying VOC concentrations depends on the used active material. Moreover, the sensor behaviors might be slightly altered when they were tested to the breath samples from different patients, although they were from the same group (either positive or negative COVID-19). This occurrence could be understood because the content and complexity of the exhaled VOCs are diverse, as discovered in another breath analysis study using GC–IMS^24^. Several VOC biomarkers could be identified as the discriminants for distinguishing between positive and negative COVID-19 patients (e.g., ethanal, acetone, acetone/2-butanone cluster, 2-butanone, methanol monomer and dimer, octanal, feature 144, isoprene, heptanal, propanol, and propanal)^24^. Nonetheless, the compounds observed from two different hospitals (i.e., Edinburgh, the United Kingdom (UK), and Dortmund, Germany) in their study were dissimilar for the same case of COVID-19 patients, which then added more complexity in analyzing the obtained breath data. These limitations were due to uncertainties in the instrument setup, operating conditions, and background contamination levels.

Thus far, a detailed study in those matters has not been performed. Meanwhile, another clinical GC–IMS study conducted by researchers in Beijing, China, suggested several other potential breath-borne VOC biomarkers for COVID-19 (i.e., acetone (C_3_H_6_O), ethyl butanoate, butyraldehyde, and isopropanol)^72^. They found that the decrease and increase in acetone (C_3_H_6_O) and ethyl butanoate levels, respectively, due to the changes in metabolites resulting from SARS-CoV-2 infections, are distinctive for COVID-19 patients^72,73^. Moreover, the average measured isopropanol and butyraldehyde for the COVID-19 patients were lower than those for the healthy control and lung cancer and non-COVID-19 respiratory infection patients. The metabolomics of exhaled breaths in critically ill COVID-19 patients were also investigated from a research consortium in France using a proton transfer reaction quadrupole time-of-flight mass spectrometer^74^. They observed four prominent VOCs (i.e., methylpent-2-enal, 2,4-octadiene, 1-chloroheptane, and nonanal) that could discriminate between COVID-19 and non-COVID-19 acute respiratory distress syndrome patients^74^. Overall, the reported MS studies in several different countries (i.e., UK, Germany, France, and China) indicate that the distinctive VOC biomarkers for COVID-19 may vary across the world and should be further investigated based on the community, race, and cases with large cohorts^75^.

In contrast to the MS method that attempts to quantitatively find and identify the exact VOC biomarkers from exhaled breaths, our technique used in GeNose C19 focuses more on the AI-based pattern analysis of integrated sensor responses to complex VOCs, qualitatively resulting from the combined extra-pulmonary metabolic and gastrointestinal manifestations of COVID-19^76^. Thus, the breath data analysis and decision-making procedure can be performed in a simple way and short time, respectively, with a high detection accuracy. To enable this, besides having a high sensitivity, chemoresistive sensors should ideally be designed to possess a high selectivity to a specific analyte in a gas mixture and zero cross-sensitivity to other compounds in the operating background. Such sensors were normally constructed in hybrid organic/inorganic structures with 3D nano-architectures (e.g., nanofibers, nanowires, and nanofins), enhancing the active surface-area-to-volume ratios^77,78^. Here, the surfaces of semiconductor nanostructures were often functionalized with certain self-assembled monolayers or polymers to specifically detect the target gas molecules^32,34,79^. Nevertheless, these organic materials suffer from low robustness. They are all well-known to degrade within a short duration of use (i.e., their chemical compositions will alter downgrading the sensor performance). As a result, pure inorganic materials (metal oxide semiconductors) are still preferably manufactured by sensor companies and widely used in gas sensing applications, including in the GeNose 19 system. Here, a single sensor alone is not sufficient for performing a specific breath pattern recognition because exhaled VOCs might have similar characteristics. This selectivity drawback could be alleviated by employing an array of 10 sensors with different sensitivities and integrating the machine learning-based breath pattern recognition algorithms.

Furthermore, to demonstrate the proof of concept ability of GeNose C19 for detecting VOCs in human breaths, we performed additional sensing assessments for acetone vapors in a modified test setup (see Supplementary Fig. 2). However, COVID-19 itself cannot be detected by simply sensing or measuring the acetone alone. This testing was mainly dedicated to demonstrate that the GeNose C19 sensor array can detect one of the VOCs normally contained in human breaths and exhibits different sensitivity levels when exposed to various gas concentration levels, which also mimics the real case of exhaled breaths from different persons or patients. The gas sensing configuration for the acetone testing, which utilizes a microsyringe for vapor injection, has already been used in our former experiments for other VOC sensor types (e.g., nanofiber-functionalized QCMs for sensing trimethylamine and butanol gases)^35,80,81^. Acetone was chosen as a VOC model in this additional study because it is not only produced in the rebreathed breath (0.8 to 2.0 ppm)^82^, along with other VOCs (alcohol) and CO, but is also one of the significant breath-borne COVID-19 biomarkers based on the study by Chen et al.^72^. Moreover, in clinical practices, breath-containing acetone has been extensively examined to diagnose other diseases (i.e., lung cancer, diabetes mellitus, starvation, and ketogenic diet)^83^.

As shown in Supplementary Fig. 2b, c, the S3 and S7 sensors (or their extracted features of F3 and F7) demonstrated the poorest responses toward acetone vapors. Conversely, the S2, S8, and S9 sensors exhibited higher sensitivities than the others. The sensor output signals given by the GeNose C19 data acquisition system agree well with those measured by a calibrated digital voltmeter. Increasing the acetone vapor concentrations from 0.04 to 0.1 µL with 0.02 intervals resulted in higher responses of the three sensors (S2, S8, and S9), whereas the S3 and S7 sensors were irresponsive (see Supplementary Fig. 2d). In particular, each vapor concentration was measured 10 times to acquire quantitative results. Lastly, as depicted in Supplementary Fig. 2e, LDA discriminated the output voltages produced by the sensors during their exposures to four different acetone concentrations (i.e., 0.04–0.1 µL).

In terms of ambient conditions, temperature and humidity might influence the performances of metal oxide semiconductor sensors^84^. Thus, to investigate their effect, we also performed cross-sensitivity assessments in respect to the two parameters for all the employed GeNose C19 sensors (see Supplementary Figs. 3 and 4). This testing is important because depending on the sensitivities of the sensors toward temperature and humidity, the obtained sensor results during the breath analysis can be disturbed, leading to a difficult interpretation of the data. Moreover, if the sensors are too reactive to the two ambient parameters, the measured data can then be unreliable to analyze the effect of VOCs in the human breath because changes in the signals were mainly affected by the temperature and humidity, not the target gases. Such a cross-sensitivity is a common reliability test for gas sensors. For GeNose C19, the environmental effect can be minimalized and controlled by performing two main procedures. First, environmental checking needs to be conducted while placing GeNose C19 in the measurement room/area. Here, the selection of the machine placement (analysis on air circulation, humidity, and temperature) plays a key role in maintaining good-quality results. GeNose C19 could sense the environmental humidity and temperature levels by utilizing humidity and temperature sensors integrated inside the system. The measurement was displayed in the program interface. Hence, the user or operator could notice the condition. In a real situation during breath sampling, the machine could only be operated if the humidity and temperature inside the chamber were in the ranges of 30–50% and 26–42 °C, as defined by the AI-based program in the system. Such a setting is adjustable to meet future demand and placement environments. Second, after checking the environmental condition, the baseline normalization protocol during the sample analysis can be done (see Methods on the GeNose preconditioning). During the AI interpretation of the VOC patterns, several protocols were employed, including signal baseline normalization. By performing baseline normalization, all the sensors that behaved and started from different baselines in different environments can always be calibrated to the standard normalization. Hence, the adaptability of the machine can be improved in new foreign environments.

In the case of acetone testing, the sensors yielded similar responses from three repeated measurements, indicating their reliable sensing results. The sensor resistance decreased (i.e., a higher output voltage was obtained) when the temperature was ramped up from 40 °C to 46 °C, and the humidity was kept stable at (30% ± 1%) RH (see Supplementary Fig. 3). Different from silicon micromechanical resonant sensors that have frequency shift interferences caused by the temperature-induced Young’s modulus change (material softening)^37,85^, the resistance decrease in the employed metal oxide semiconductor sensors (e.g., *n*-type SnO_2_ with a bandgap of 3.6 eV) at high temperatures was caused by the increasing number of electrons that have sufficient energy crossing to the conduction band and thus contributing to the conductivity^86^. Because this is a natural characteristic of semiconductor materials, we could overcome this effect in GeNose C19 by controlling the temperature inside the test chamber at relatively stable values (i.e., (42 °C ± 2 °C)) during the sensing phase of the exhaled breath.

Similar to the trend shown in the cross-temperature test, the sensor resistance also dropped to a lower value, resulting in a higher output voltage when the relative humidity was raised from 30% to 35% and the temperature was set constant at (40 °C ± 1 °C) (see Supplementary Fig. 4). The electrical characteristics of metal oxide semiconductors changed due to the water adsorption on their surface while being exposed to humid air. Two different mechanisms of chemisorption and physisorption processes took place to create the first layer (i.e., chemisorbed layer) and its subsequent films of water molecules (i.e., physisorbed water layers), respectively^87^. If the first chemisorbed layer has been formed, then the successive layers of water molecules will be physically adsorbed on the first hydroxyl layer. Because of the high electrostatic fields in the chemisorbed layer, the dissociation of physisorbed water can easily occur, producing hydronium ion (H_3_O^+^) groups. Here, the conduction mechanism relies on the coverage of adsorbed water on the metal oxide semiconductor. First, in the event only hydroxyl ions exist on the metal oxide surface, the charge carriers of protons (H^+^) resulting from hydroxyl dissociation will hop between adjacent hydroxyl groups. Second, after the water molecules have been adsorbed but not fully covered the oxide surfaces, the charge transfer will be dominated by H_3_O^+^ diffusion on hydroxyl groups, despite the occurring proton transfer between adjacent water molecules in clusters. Finally, once the continuous film of the first physisorbed water has been formed (i.e., full coverage of metal oxide by the physisorbed water layer), proton hopping between neighboring water molecules in the continuous film will be responsible for the charge transport^88^. More detailed explanations of the sensing mechanism and adsorption of water molecules on metal oxide semiconductor surfaces are described elsewhere^84,87,88^. Again, in the conducted cross-sensitivity measurements (Supplementary Fig. 4), the signal changes of the GeNose C19 sensors affected by humidity are relatively lower (i.e., <100 mV) compared to those exposed to exhaled breaths (i.e., ~1 V, as shown in Fig. 3a, b). Thus, temperature and humidity will insignificantly influence the system performance during breath measurements, when GeNose C19 has been well preconditioned.

To confirm the performance of our GeNose C19, RT-qPCR was used as the reference standard on the basis of the health service standard protocol underlined by the Indonesian Ministry of Health. Based on the analysis of the RT-qPCR protocol using Bayes’ theorem, RT-PCR tests cannot be solely relied upon as the gold standard for SARS-CoV-2 diagnosis at scale. Instead, a clinical assessment supported by a range of expert diagnostic tests should be used. Here, although our study mentioned that RT-qPCR was used as the reference standard, clinical data from each patient were also collected and analyzed.

According to a recently published systematic review study, the need for repeated testing in patients with suspicion of SARS-Cov-2 infection was reinforced because up to 54% of COVID-19 patients might have an initial false-negative RT-qPCR^89^. Meanwhile, in the case of false-positive rates of RT-qPCR, much lower values (i.e., 0–16.7% with an interquartile range of 0.8–4.0%)^90,91^ were exhibited in several studies, which were affected by the quality assurance testing in laboratories. Public Health England also reported that RT-qPCR assays showed a specificity of over 95%, so up to 5% of cases were false positives^91^. Moreover, the overall false-positive rate of high throughput, automated, sample-to-answer nucleic acid amplification testing on different commercial assays was only 0.04% (3/7,242, 95% CI, 0.01% to 0.12%)^92^. False-positive SARS-CoV-2 RT-qPCR results could originate from different sources (e.g., contamination during sampling, extraction, PCR amplification, production of lab reagents, cross-reaction with other viruses, sample mix-ups, software problems, data entry errors, and result miscommunication)^93^. In our case, all the bought and used reagents were checked and calibrated daily to avoid false positives (i.e., no false positive of RT-qPCR result was found in this study). Meanwhile, the false-negative of the RT-qPCR result was found in three patients in their first examination, but positive results were revealed on the second examination the next day. Again, the detailed test procedure can be found in the Methods.

Currently, diagnostic methods used to screen COVID-19 are antigen test, rapid molecular test, and chest CT scan. Antigen tests have an average sensitivity of 56.2% (95% CI: 29.5–79.8%) and average specificity of 99.5% (95% CI: 98.1–99.9%)^94^. The average sensitivity and specificity for the rapid molecular tests are 95.2% (95% CI: 86.7–98.3%) and 98.9% (95% CI: 97.3–99.5%), respectively^94^. Meanwhile, chest CT scan possesses an average sensitivity and specificity of 87.9% (95% CI: 84.6–90.6%) and 80.0% (95% CI: 74.9–84.3%), respectively^95^. Nonetheless, these diagnostic methods have their drawbacks. The average sensitivity of antigen tests is not high, as shown by the study above, and it declines when the viral load decreases, which often happens to COVID-19 patients. Moreover, the sample collection is invasive (by a nasopharyngeal or oropharyngeal swab). Rapid molecular testing also employs an invasive sample collection method (by a nasopharyngeal or oropharyngeal swab), and the turnaround time of point-of-care rapid molecular tests still takes at least 20 min^96^. Moreover, chest CT scan exposes patients to radiation and is not specific.

Compared to these diagnostic methods, GeNose C19 has the potential to be a screening test. A breath test with the portable GeNose C19 is noninvasive and easy to use because it only requires patients to breathe into a sampling bag with minimal preparation, has a fast analysis time, and does not have radiation concerns. Similar to other biological samplings in several laboratory examinations (e.g., blood glucose sampling and chemical blood analysis), GeNose C19 also requires preparation of subjects before breath sampling, such as fasting (i.e., refraining from eating, smoking, or drinking anything other than water at minimum 1 h before sampling). However, the duration of the analysis starting from the breath sampling to the test result decision only takes ~3 min. The sensitivity and specificity results of GeNose C19 from the profiling tests show that combining GeNose C19 with an optimum machine learning algorithm can accurately distinguish between positive and negative COVID-19 patients. Hence, it opens an opportunity for using this developed breathalyzer as a rapid, noninvasive COVID-19 screening device based on exhaled breath-print identification.

Several factors may influence breath-prints, i.e., pathological and disease-related conditions (smoking, comorbidities, and medication), physiological factors (age, sex, food, and beverages), and sampling-related issues (bias with VOCs in the environment)^97^. A previous study revealed that older age altered breath-prints in patients with lung cancer^98^. There were concerns that several other respiratory diseases may present similar VOC patterns to those from the COVID-19. Several studies reported that several comorbid and confounding factors (e.g., chronic obstructive pulmonary disease, asthma, tuberculosis, and lung cancer) might affect the composition of VOCs^99,100^. Thus, patients with other respiratory diseases can have different patterns of VOCs that result in different sensor signals, suggesting that the electronic nose may still determine the COVID-19 infection to a certain degree by continuing to train its AI database in reading VOCs from confirmed positive COVID-19 patients. Our studies showed no significant difference in the detected sensor signal patterns of patients with comorbidities compared to those without comorbidities. Nonetheless, due to the few comorbid cases obtained in our subjects, which could be considered the limitation in our current study, the influence of existing comorbidities on the VOC pattern cannot be concluded and will be further evaluated in the next research.

Food and beverages (e.g., poultry meat and plant oil) can influence breath-prints, whereas smoking may increase the levels of benzene, 2-butanone, and pentane and simultaneously decrease the level of butyl acetate in exhaled breaths^101–103^. In our study, none of the patients was smoker. The comorbidities were also comparable between the case and control groups. There was no significant difference in the consumption of food and beverages between the two groups. The measurements were conducted in the same environment for all the participants. Thus, there was no bias with other interfering VOCs.

However, the possible presence of physiological variations resulting from physiological and biochemical changes in the body due to alterations in the respiratory rhythm affected by the manipulated breathing technique should also be considered^61^. Therefore, in our work, breath sampling was performed in such a defined protocol to collect only the third exhaled end-tidal breath. Accordingly, the natural breathing pattern and rhythm can be preserved, resulting in minimal changes in VOCs. We avoided excessive effort or repeated sampling in each breath collection as previous studies reported that it might alter the quality of collected VOCs^104^. The disturbance from other factors to breath test results is minimal. However, such confounding factors are most likely present in the real implementation and can affect at least breath-prints to a certain degree. Further study is now being conducted to reveal the effects of various confounders.

Our study using GeNose C19 did not evaluate the distinctive concentration of each VOC found in breath samples of patients with positive or negative COVID-19. However, to investigate the types of VOCs produced in exhaled breaths of the positive and negative COVID-19 patients, we conducted another characterization based on GC–MS for several exhaled breaths of patients (see Supplementary Table 3). In the extracted results, there was no significant difference in the composition of VOCs between patients with positive and negative COVID-19, suggesting that the difference in the breath-print pattern may be contributed by the variation in the concentration or proportion of several VOCs rather than the presence of one or two signature VOCs. For example, acetone was reported to be one of the VOCs with the highest concentration emitted by healthy humans^104^. However, in COVID-19-positive patients, acetone was reported to be in a lower proportion, compared to the healthcare worker or healthy control group^72^. Meanwhile, another VOC (i.e., ethyl butanoate) has been reported as one of the signature VOCs in COVID-19 patients, whose concentration is slightly higher compared to the healthy control^72^.

Anosmia (i.e., the olfactory system cannot accurately detect or correctly identify odors) is one of the most frequently identified COVID-19 symptoms^45,105^. CO has been linked with this issue because it is an olfactory transduction byproduct related to the reduction of cyclic nucleotide-gated channel activity that causes a loss of olfactory receptor neurons^45,106^. In our GC–MS results (Supplementary Table 3), six sensors in GeNose C19 (i.e., S1, S3, S4, S5, S6, and S8) could detect CO. Aside from CO, the GC–IMS studies in Dortmund, Germany, and Edinburgh, UK indicated that aldehydes (ethanol and octanal), ketones (acetone and butanone), and methanol are biomarkers for COVID-19 discrimination^24^. This result is however different from the finding from another research group in Garches, France, using the proton transfer reaction quadrupole time-of-flight MS, where four types of VOCs (i.e., 2,4-octadiene, methylpent-2-enal, 1-chloroheptane, and nonanal) could discriminate between COVID-19 and non-COVID-19 acute respiratory distress syndrome^74^. Studies conducted in two cities in the USA (Detroit, Michigan and Janesville, Wisconsin) by Liangou et al. reported another set of eight compounds (i.e., nitrogen oxide, acetaldehyde, butene, methanethiol, heptanal, ethanol, methanol-water cluster, and propionic acid) as key biomarkers for the COVID-19 identification in human breath. Moreover, in Leicester, UK, seven exhaled breath features (i.e., benzaldehyde, 1-propanol, 3,6-methylundecane, camphene, beta-cubebene, iodobenzene, and an unidentified compound) measured by the desorption coupled GC–MS were employed to separate RT-qPCR-positive COVID-19 patients from healthy ones^107^. In our measurement, camphene was detected only in the negative COVID-19 breath sample by S10.

Furthermore, Chen et al. reported two sequential GC–MS studies in Beijing, China, that resulted in totally different breath-borne biomarkers for COVID-19 screening, despite using the same measurement approach^72,108^. Their first measurement reported in 2020 indicated that COVID-19 and non-COVID-19 patients could be differentiated by solely monitoring three types of VOCs (i.e., ethyl butanoate, butyraldehyde, and isopropanol)^72^. Nonetheless, in their second report in 2021, acetone was detected as the biomarker among many VOC species because its levels were substantially lower for COVID-19-positive patients than those of other conditions^73^. In our GeNose C19 sensor array, acetone can be detected in S8^109^. Recently, ammonia has also been proposed as another biomarker for COVID-19, whose relation to complications stemming from the liver and kidneys was affected by the SARS-CoV-2 infection^110^.

In all the already described examples of MS studies worldwide, the identification and determination of specific COVID-19 biomarkers in breath clearly remain challenging. Here, different discriminant compounds can be yielded depending on several parameters (e.g., measurement technique, filtering approach, location, and breath sample type). Nonetheless, we can still extract some information from our GC–MS results (Supplementary Table 3). A hydrocarbon of ethylene was sensed by S10 in the positive COVID-19 breath sample. Meanwhile, for the negative COVID-19 breath samples, other hydrocarbons (i.e., butyl aldoxime, decane, and benzene) were detected by S10. Furthermore, S2 and S9 could measure a few specific esters (i.e., benzoic acid, 3-hydroxymandelic acid, and acetic acid) in the negative COVID-19 samples. Generally, the appearances of the three sensors (S2, S9, and S10) were dominant as compared to those of the others. For instance, S2 and S9 were highly sensitive toward aldehydes and esters, whereas S10 was likely to be reactive toward hydrocarbons.

Regardless of the successful compound extraction and its association with GeNose C19 sensor array, our GC–MS characterization was only performed in a low number of samples. Therefore, a further investigation with a larger number of breath samples still requires to be carried out in the near future to correlate the measurement results of GeNose C19 and GC–MS methods in a more thorough way, especially in Indonesia. This method also includes more investigations on the possible influence from other respiratory-related viruses (e.g., influenza, respiratory syncytial virus, and rhinovirus). The presence of viruses other than SARS-CoV2 will affect the VOC profile in breaths to a certain degree. However, in our current setup, it will be mostly recognized by the AI algorithm in GeNose C19 as “non-reactive,” which means that it contains VOC-based breath-prints not typical to a SARS-CoV2 infection. Influenza and rhinovirus infections manifest a high amount of heptane, nitric oxide, and isoprene^111^. Consistently, our preliminary study on breath samples from a few patients confirmed to have rhinovirus based on RT-qPCR and showed a high response on S8, suggesting a high amount of isoprene or isopropanol. However, further comparison analysis with more numbers of validated breath samples data will be definitely necessary to obtain a solid conclusion on this matter.

In terms of the enhanced sensing technology, once the VOC biomarkers can be clearly determined, a molecular imprinting method could be applied to generate highly selective sensors that target these specific VOC markers. Hence, the sensitivity and specificity of GeNose C19 and its overall accuracy can be further improved. Another critical step for the system development is to conduct a diagnostic test with a large cohort to strongly elucidate its potential as a diagnostic tool in the near future.

Other limitations of our study are that a direct correlation between the level of the virus gained from the swab and the amount of VOC concentration could not be drawn. These conditions are partly caused by the fact that VOCs were not directly produced by the virus, but rather by host cells infected by the virus as a part of their metabolic response to the infection. GeNose C19 could only predict the presence of the virus based on the resulting VOCs in the breath produced by respiratory tract epithelial cells and immune cells that were infected by the SARS-CoV2 virus. Nevertheless, a study on the correlation between the positivity rate of breath results and level of the cycle threshold value (Ct value) gained from RT-qPCR examination has been of interest for the next research. Here, more insights into the performance of GeNose C19 will be gained in terms of sensitivity, specificity, and accuracy levels correlated with the level of Ct value of RT-qPCR. The Ct value itself is currently accepted as an alternative parameter to determine the level of the viral load in each individual on the basis of the minimum cycle threshold necessary to duplicate the viral component to be read. Nonetheless, GeNose C19 combined with RT-qPCR using the Ct value has a limitation for estimating the exact number of the viral load. It was also a question of whether a person with a positive PCR test result for SARS-CoV-2 is automatically infectious or infectious only if the Ct value is below a certain limit (e.g., Ct value of <35)^112,113^. In another study, knowing the typical viral load of SARS-CoV-2 in bodily fluids and host tissues, the total number and mass of SARS-CoV-2 virions in an infected person could be estimated^114^. Each infected person carries the total number and mass of SARS-CoV-2 virions of 10^9^–10^11^ virions and 1–100 μg, respectively, during the peak infection^114^.

Again, this study was meant to demonstrate a proof of concept that breath sampling and detection can be used to predict COVID-19 infection. Essentially, the calculated performance values in our study show the reliability of the DNN algorithm in predicting the training and testing data of breath samples, suggesting the great potential of the GeNose technology, fortified by the DNN algorithm to be used as a COVID-19 screening tool. Here, we performed the study using a so-called open-label design, where we already knew the COVID-19 status of the subjects before conducting sampling and classifying the sampled data into case and control groups. Using this method, we read, found, and compared the breath sample pattern profiles in each respected group and employed them as training data to build our first AI database, in which all data were validated by the test results of RT-qPCR supported with clinical data. A combined measurement of GeNose C19 with GC–MS will be conducted in the near future to answer questions related to distinctive VOCs for COVID-19.

Lastly, another critical step for the system development is to confirm the usability and performance in the clinical setting, where a study on the clinical diagnosis of COVID-19 with a larger number of exhaled breath samples is currently performed to prove the potential of GeNose C19 as a rapid COVID-19 screening tool using a cross-sectional design and double-blind randomized sampling. Here, breath samples and nasopharyngeal swab specimens are taken in the situation where the operator or sampler does not know the “true” condition of patients. A double-blind fashion means that neither the sampler nor subjects know their true condition during the sampling process. The breath samples were analyzed by GeNose C19 without knowing the result of RT-qPCR, and swab samples were examined by RT-qPCR without prior knowledge of the GeNose C19 result. Both results were then compared to each other to draw a conclusion. In this approach, RT-qPCR will still be used as the reference standard.

## Methods

### Ethical statement

This study was approved by the Medical and Health Research Ethics Committee of the Faculty of Medicine, Public Health and Nursing, Universitas Gadjah Mada/Dr. Sardjito General Hospital, Yogyakarta, Indonesia, with reference number KE/0489/05/2020, and had been registered in clinicaltrials.gov (NCT04558372)^115^. All procedures were conducted following the relevant guidelines and regulations based on the Good Clinical Practice and the Helsinki Declaration of 2013^116^.

### Study design and patient recruitment

The study was designed as an open-label case-control prospective cohort study. The healthcare workers and patients were informed regarding the given treatment. Subjects were consecutively recruited, and written informed consent was obtained from the subjects and their guardians (for subjects aged 5 to 17 years) before admission. Subjects, who were eligible in the study, were classified into two groups (i.e., case and control groups), as depicted in Fig. 4. The case group consisted of patients with confirmed COVID-19, who were admitted to the isolation rooms of two hospitals in the Special Region of Yogyakarta, Indonesia (i.e., Bhayangkara General Hospital in Sleman District (RS Bhayangkara) and Bambanglipuro COVID-19 Special Field Hospital in Bantul District (RSLKC Bantul)).

COVID-19 infection was confirmed by the RT-qPCR tests on the SARS-CoV-2 RNA extracted from their nasopharyngeal and oropharyngeal swabs. The swab samples were taken in the respective hospitals using protocols in accordance to the World Health Organization (WHO) and Centers for Disease Control and Prevention (CDC) standards and sent to a certified national laboratory in Indonesia (i.e., *Balai Besar Teknik Kesehatan Lingkungan dan Pengendalian Penyakit* and Laboratory of COVID-19, Faculty of Medicine, Public Health and Nursing, Universitas Gadjah Mada, Yogyakarta) for RT-qPCR tests. Primers and reagents used in the RT-qPCR examination were determined and provided nationally by the Indonesian Ministry of Health’s regulation. The total viral RNA was extracted using a QiAMP Viral RNA mini kit (Qiagen, Hilden, Germany). SARS-CoV-2 was then detected using Real-Q 2019-nCoV Detection Kit (BioSewoom, Seoul, South Korea, targeting the *RdRp* and *E* genes) with LightCycler 480 Instrument II (Roche Diagnostics, Mannheim, Germany). The RT-qPCR examination was performed within three to four days after the initial suspicion of having contact with another confirmed COVID-19-positive person, regardless of whether the patient exhibited symptoms or not, in accordance to the COVID-19 healthcare service guideline established by the Indonesian Ministry of Health.

To minimize the possibility of having false negatives, RT-qPCR was performed twice in our experiment. If the first result was negative, then the second examination was conducted on the next consecutive day. If both examinations showed negative results, then the patient was confirmed to be negative of COVID-19. Meanwhile, to avoid or minimize the probability of obtaining false positives, the RT-qPCR testing has been guaranteed to be performed based on the established protocols using the standardized reagent kit determined by the Indonesian Ministry of Health’s regulation and performed by trained laboratory professionals in a biosafety level 2 laboratory, where the machine is calibrated daily to avoid any contamination, including cross-contamination with other samples.

Subjects who had worsening clinical symptoms and were unable to provide breath samples were excluded from the study. All COVID-19-positive patients admitted to the isolation room received standard treatment based on the WHO guidelines. The control group consisted of patients admitted to the hospital with diseases other than COVID-19 or being suspected to have close contact with positive cases during mass screening. The patients in the control group possessed negative RT-qPCR examination results that were tested two times.

### Medical treatment

All clinical data were recorded daily, including anthropometric data, laboratory results, and chest X-ray images. All patients received medical treatment according to the WHO and standard treatment guidelines in hospitals.

### GeNose C19 preconditioning

Preconditioning of the GeNose C19 system needs to be performed before its daily usage because this developed sensor system possesses several limitations, especially related to other interfering odor sources. Thus, several points designated as a standard operation procedure should be followed to ensure a stable and proper working of the equipment to sniff out COVID-19. First, a decent fresh-air circulation should be assured during the operation of GeNose C19 in indoor and outdoor environments. Second, as the sensing system comprises thermally activated semiconductor metal oxide sensors (S1–S10), it needs to be turned on and preheated for at least 30 min before its operation to obtain stable signal baselines. Each sensor was integrated with an internal heater, enabling the activation temperature at a few hundred degrees Celsius. Third, the gas monitoring chamber must be cleared away from the possible rest contaminants and previously tested VOCs by selecting the “flushing mode” for 30 min. Here, the chamber was circulated with reference air from the ambient. Fourth, the patients should neither eat nor drink anything other than water for 1 h before exhaling their breath to air sampling bags. Short fasting assists GeNose C19 to record an accurate measure of the sensor signal pattern. Finally, the surrounding environment should not possess excessive interfering odor sources. The system needs to be analyzed in the absence of a sampling bag, in which the sensor signal increase should not be more than 400 mV during the “analyze mode.” If the signal level reaches above this value, then GeNose C19 has to be moved to other more suitable and cleaner places.

The GeNose C19 machines were calibrated and adjusted during production and after being out-delivered from the factory. In terms of hardware, machine variability has been guaranteed by the quality control procedure to ensure low tolerance within different machines. In this study, we used two machines that were pre-calibrated before deployment. Pre-deployment calibration was performed on each sensor using standardized gases. As the calibration update is important for the practical use of the electronic nose comprising metal oxide semiconductor sensors, several methods can be applied to tackle issues of temporarily sensor drifts, matrix effects, and calibration transfers between instruments. The calibration methods include multivariate calibration update, adaptive drift correction, sample transfer, component correction, and direct standardization^117^. Moreover, a wide number of algorithms (including various machine learning models) can be utilized to support drift reduction and calibration update^117–120^.

### Exhaled breath sampling procedure

Breath samples were collected from two different patient groups (i.e., RT-qPCR-confirmed positive and negative COVID-19) using a modified single-use non-rebreathing mask connected to sampling bags (see Fig. 2). Breath samples were taken every day during hospitalization until the day of discharge. Patients in both groups (positive and negative COVID-19) were situated in different rooms. Positive COVID-19 patients were admitted in the isolation room with an active separated exhaust system, whereas negative COVID-19 patients were admitted to the common ward. We applied a safe breath collection procedure to ensure the safety of healthcare workers and other patients.

The collectors (nurses) who previously received specialized and standardized training for breath sampling were evaluated by Cohen’s kappa method to assess their agreement procedure^121^. In addition, they wore personal protective equipment (level 3) for COVID-19 according to the standards and recommendations from the WHO, which include respirator N95, gown, gloves, eye protection (face shield), and apron^122^. The subjects were instructed to take two initial normal breaths and exhale per usual using their protective masks. Afterward, they were requested to exhale their third breath, in an end-expiratory volume breath fashion^60^, into the mask-integrated sampling bag entry hole. The sampling bag was subsequently sealed and connected to the GeNose C19 machine via the collecting hose and HEPA filter (see Fig. 2c). Before being analyzed by the electronic nose, the breath sample was purified by a commercial HEPA filter through a PTFE medical tube. The invalid breath samples were excluded from the data processing.

### Pattern recognition algorithms

Before being analyzed by machine learning models, the obtained gas sensor array responses were subtracted from their initial baseline values, and their features in the time domain were then extracted by four parameters, i.e., maximum, median, standard deviation, and variance values (see Fig. 3 and Supplementary Fig. 7). The four parameters were obtained from the signals of 10 sensors to generate 40 features that were fed into the machine learning models. A similar feature extraction method was applied in other reported electronic nose systems^123^. It was considered one of the simplest yet reliable methods among many extraction method options. Moreover, we selected the four features because of several reasons: First, maximum values could represent the highest VOC concentration interacted with the sensors. Second, median values were used to stabilize the machine learning if outliers were noticed. Third, standard deviation and variance values were gathered to improve the precision in each algorithm used. Thus, the more data collected, the better the performance of the algorithm.

Pattern recognition was applied not only to obtain relevant data but also to eliminate redundant information. Hence, the classification model performance can be increased. In this study, the four feature extraction parameters were employed as input parameters for the machine learning models, in which four classification algorithms (i.e., LDA, SVM, MLP, and DNN) were assigned. The ROC graphs were also added to yield the sensitivity (true-positive ratio function) and specificity (true-negative ratio function) plots for all the parameter points. Each point in a ROC curve represents the coupled sensitivity/specificity related to a particular boundary decision. The AUC values were determined as a performance measure for machine learning algorithms related to their abilities in differentiating between positive and negative COVID-19 groups. The hybrid learning method based on feature clustering and scoring can be employed to further enhance the COVID-19 breath analysis by an electronic nose^109^.

All datasets were divided into two groups (i.e., training and testing data) to evaluate each classification algorithm's performance. The groups were randomly split with ratios of 70% and 30% for training and testing data, respectively. Ten-fold cross-validation was also employed to prevent overfitting. Moreover, LDA, which could be performed without changing the parameters, acted as a baseline model. The SVM model has several significant parameters determining its classification performance (e.g., cost value, gamma value, and kernel function). A grid-search procedure was applied to choose the optimum SVM parameters. A TPOT library was also utilized to find the finest combination of MLP parameters using a genetic algorithm, in which 100 generations, 100 population size, 0.9 mutation rate, and 0.1 cross-over rate were applied in the computation. Furthermore, based on several layers having connected neurons, a DNN with feed-forward and back-propagation algorithms was attempted to be applied in the GeNose C19 data analysis. Here, the main hyperparameters (e.g., number of hidden layers and neurons, activation function, dropout layer, and optimizer function) were involved during data processing to yield the optimum model.

Among the 615 breath samples (i.e., 330 positive and 285 negative COVID-19 breath samples), 70% were used as training data, whereas the remaining 30% were utilized as testing data from each respective group (case or control group). Training data were used to build the models (algorithms). Later, 70% of the data were tested once more to check the reliability of the models (so-called internal validation). Meanwhile, the remaining 30% were used to test the machine learning models to evaluate their performances. In Fig. 5, the results from three different datasets are displayed (i.e., results of training, testing, and all data). The training data results, shown as the outcome of internal validity, represent the reliability and stability of the built models. The testing data results represent the accuracy and prediction results of the tested data. All data results show the prediction of combined testing and training data (100%) using the models, which were built and trained from 70% of the data (training data)^123^.

### Characterizations of the filter and VOCs

The morphology of the fiber filter was analyzed using a scanning electron microscope (JSM–6510, JEOL Ltd., Tokyo, Japan). The types of VOCs contained in the RT-qPCR-confirmed positive and negative COVID-19 breath samples were investigated by employing a GC–MS machine (ISQ 7000 single quadrupole GC–MS system, Thermo Fisher Scientific Inc., MA, USA).

## Supplementary information

Supplementary information

COE English Editing

Ethical Clearance Document

clinical trial approval letter

## Data Availability

The data that support the findings of this study are available from the corresponding authors upon reasonable request.
