# Supplementary material for: Fast and noninvasive electronic nose for sniffing out COVID-19 based on exhaled breath-print recognition
Source: NPJ Digit Med. 2022 Aug 16;5:115. doi: 10.1038/s41746-022-00661-2 (PMC9379872; doi:10.1038/s41746-022-00661-2)
Supplement: Supplementary file 1 — Supplementary information [file 41746_2022_661_MOESM1_ESM.docx]

Supplementary Information

**Fast and noninvasive electronic nose for sniffing out COVID-19 based on exhaled breath-print recognition**

**Dian Kesumapramudya Nurputra^1,2,*^, Ahmad Kusumaatmaja^3^, Mohamad Saifudin Hakim^4^, Shidiq Nur Hidayat^3,5^, Trisna Julian^5^, Budi Sumanto^3^, Yodi Mahendradhata^6,7^, Antonia Morita Iswari Saktiawati^7,8^, Hutomo Suryo Wasisto^5,*^, Kuwat Triyana^3,*^**

^1^ Department of Child Health, Faculty of Medicine, Public Health and Nursing, Universitas Gadjah Mada, Jl. Farmako Sekip Utara, Yogyakarta 55281, Indonesia

^2^ Postgraduate Program in Clinical Medicine Science, Faculty of Medicine, Public Health and Nursing, Universitas Gadjah Mada, Jl. Farmako Sekip Utara, Yogyakarta 55281, Indonesia

^3^ Department of Physics, Faculty of Mathematics and Natural Sciences, Universitas Gadjah Mada, Sekip Utara PO Box BLS 21, Yogyakarta 55281, Indonesia

^4^ Department of Microbiology, Faculty of Medicine, Public Health and Nursing, Universitas Gadjah Mada, Jl. Farmako Sekip Utara, Yogyakarta 55281, Indonesia

^5^ PT Nanosense Instrument Indonesia, Umbulharjo, Yogyakarta 55167, Indonesia

^6^ Department of Health Policy and Management, Faculty of Medicine, Public Health and Nursing, Universitas Gadjah Mada, Yogyakarta, Indonesia

^7^ Center for Tropical Medicine, Faculty of Medicine, Public Health and Nursing, Universitas Gadjah Mada, Jl. Farmako Sekip Utara, Yogyakarta 55281, Indonesia

^8^ Department of Internal Medicine, Faculty of Medicine, Public Health and Nursing, Universitas Gadjah Mada, Jl. Farmako Sekip Utara, Yogyakarta 55281, Indonesia

* Corresponding authors.

E-mails: [dian.k.nurputra@ugm.ac.id](mailto:dian.k.nurputra@ugm.ac.id) (D.K.N.); [h.wasisto@nanosense-id.com](mailto:h.wasisto@nanosense-id.com) (H.S.W.); [triyana@ugm.ac.id](mailto:triyana@ugm.ac.id) (K.T.)

Contents

[1. Pre-conditioning of GeNose C19 to ambient air S-2](#_Toc103506212)

[2. GeNose C19 assessment toward acetone vapors S-3](#_Toc103506213)

[3. Effects of ambient temperature and humidity on sensor array S-4](#_Toc103506214)

[4. Principal component analysis (PCA) S-6](#_Toc103506215)

[5. Machine learning performance analysis S-7](#_Toc103506216)

[6. Exhaled breath data processing steps S-8](#_Toc103506217)

[7. Non-parametric ANOVA test S-9](#_Toc103506218)

[8. Pairwise difference between classification models S-10](#_Toc103506219)

[9. Gas chromatography-mass spectroscopy (GC-MS) S-11](#_Toc103506220)

# Pre-conditioning of GeNose C19 to ambient air


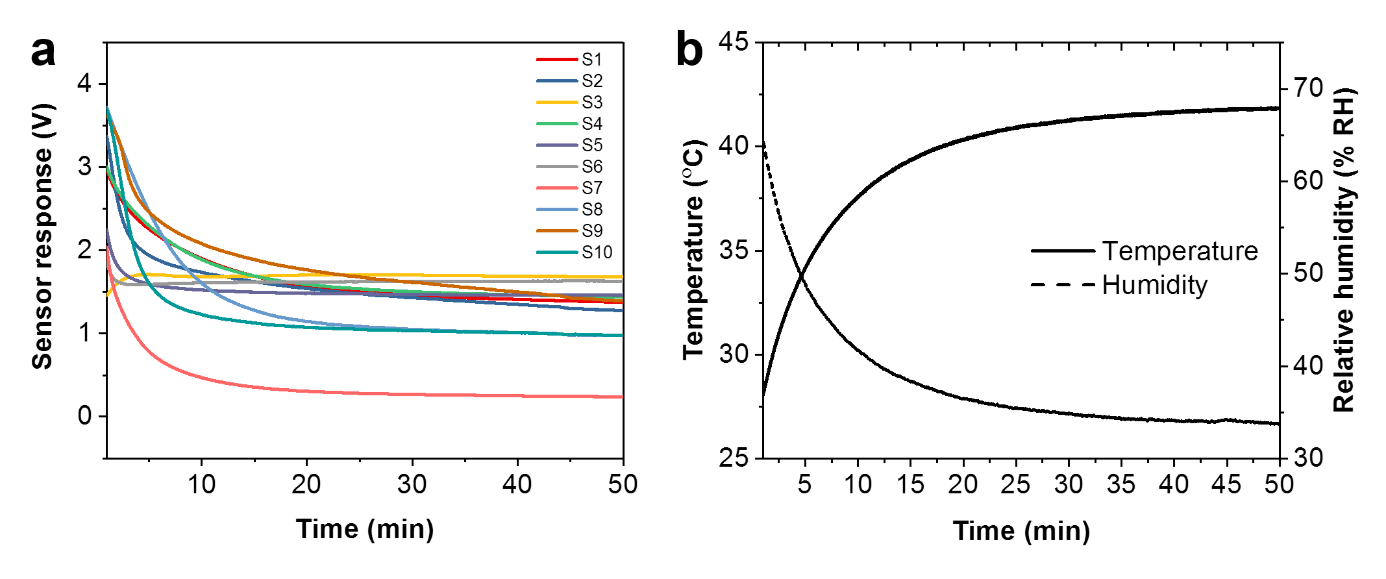


**Supplementary Figure 1.** **Pre-conditioning of GeNose C19.** **a** Real-time sensor array responses during preconditioning process where all the sensors are exposed to ambient air. **b** Measured temperature and humidity levels inside the gas chamber. All the sensors reach their stable conditions after being pre-heated for ~20 min. Each sensor possesses an internal heater that enables an activation temperature at a few hundred degrees Celsius.

# GeNose C19 assessment toward acetone vapors


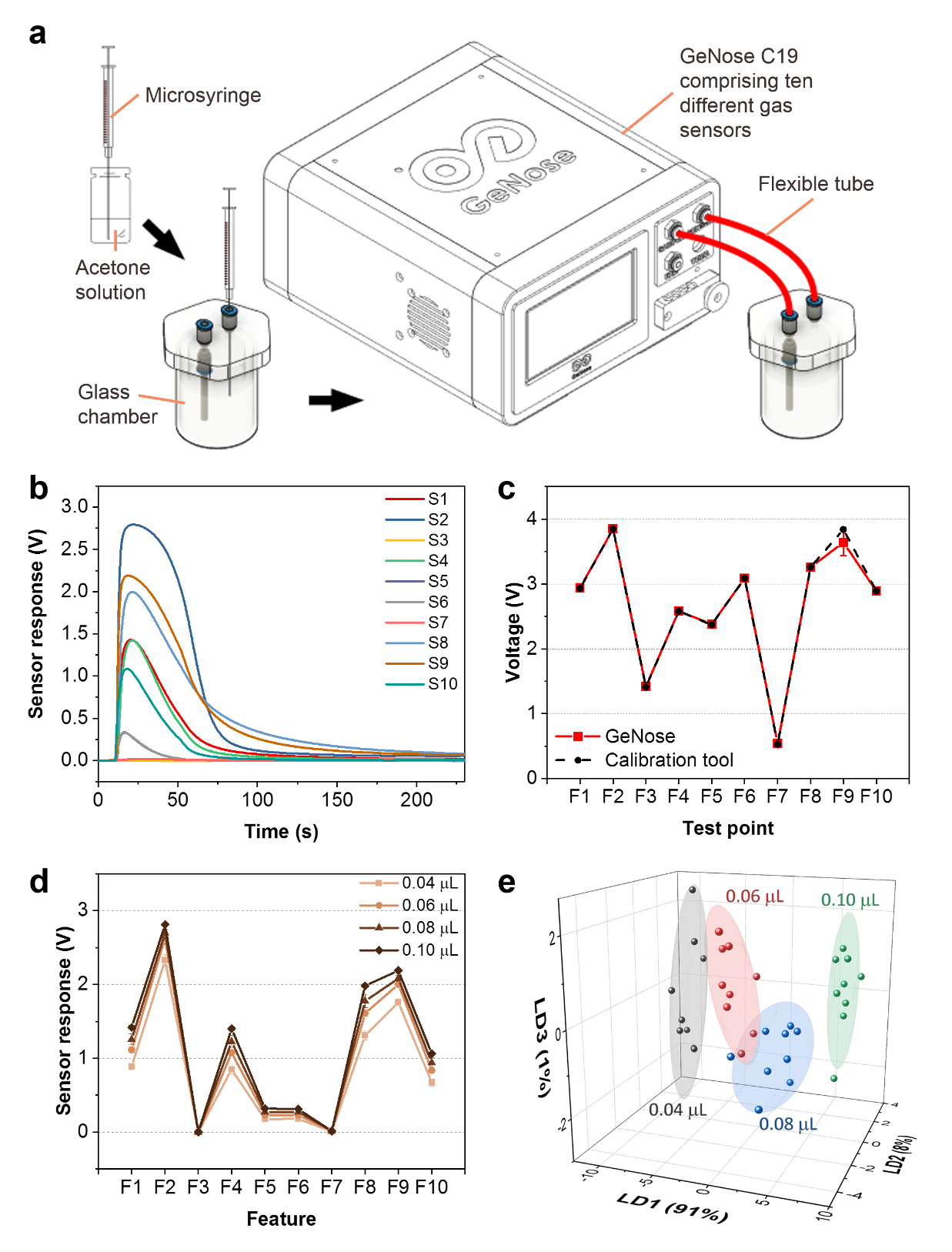


**Supplementary Figure 2.** **Exposure assessment of GeNose C19 to acetone vapors.** **a** Gas measurement setup for GeNose C19 assessment toward specific volatile organic compound (VOC). Acetone vapors were used as a VOC model, in which they were injected to the gas chamber using a 1 μL microsyringe. **b** Responses of ten different metal oxide semiconductor sensors (S1-S10) integrated in GeNose 19 system during their exposure to acetone vapors with a concentration of 0.10 µL. **c** Comparison of sensor signal readout between GeNose C19 and calibrated digital voltmeter. The extracted response features are defined as F1 – F10. **d** Sensor responses during exposure to acetone vapors with different concentrations (0.04 – 0.10 µL). Each vapor concentration was tested ten times to acquire quantitative values. **e** Classification of four different acetone vapor concentrations employing linear discriminant analysis (LDA).

# Effects of ambient temperature and humidity on sensor array


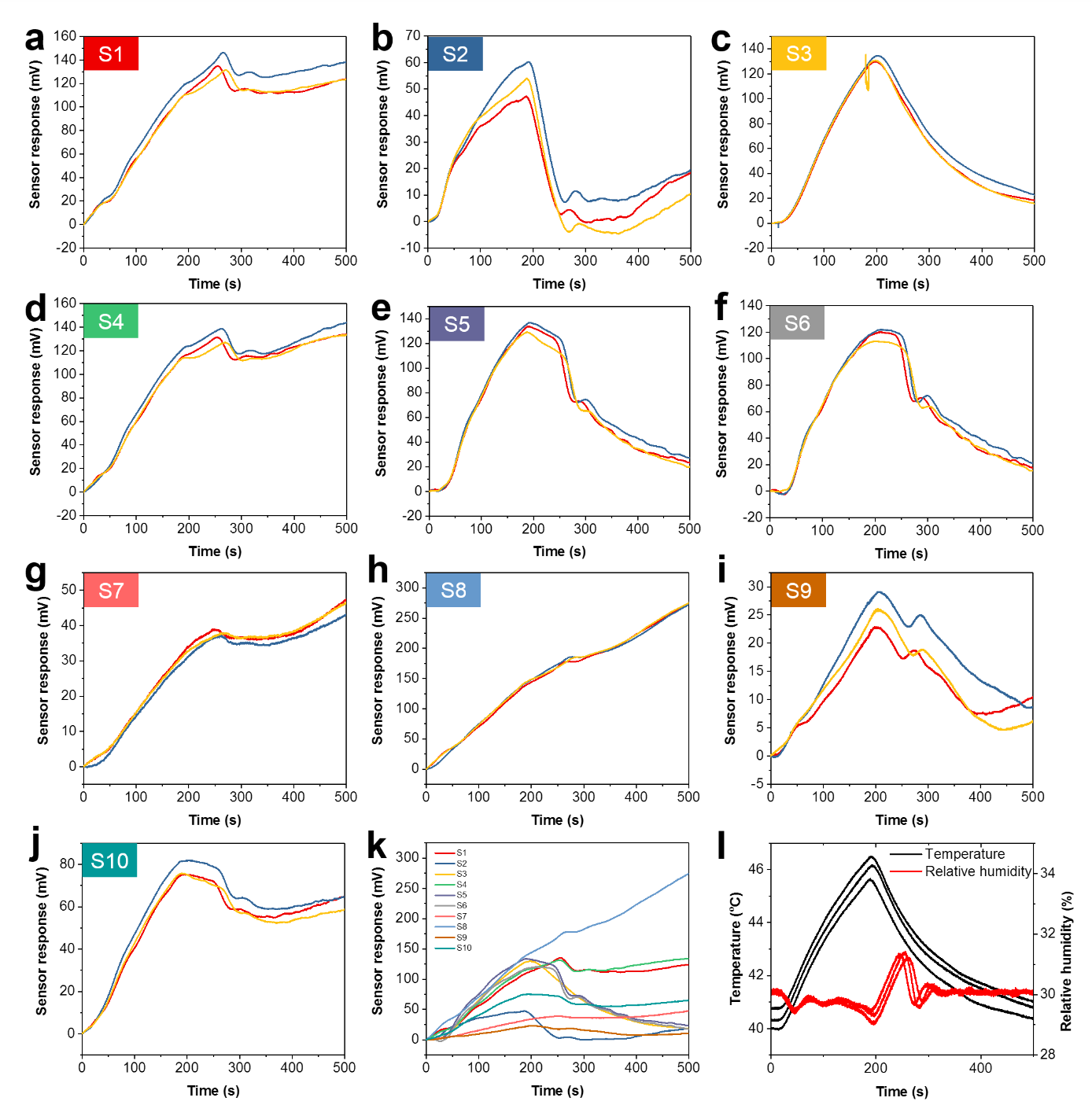


**Supplementary Figure 3.** **Cross-temperature test of GeNose C19.** **a-j** Responses of ten different gas sensors in GeNose C19 (S1 – S10) during cross-temperature tests. The temperature was modified in real time, while the humidity was kept at relative stable values. **k** Sensor array sensor signals during the first temperature test. **l** Temperature and humidity measured during cross-sensitivity assessment. From three repeated measurements, the sensors yield similar responses indicating their high sensing reproducibility.


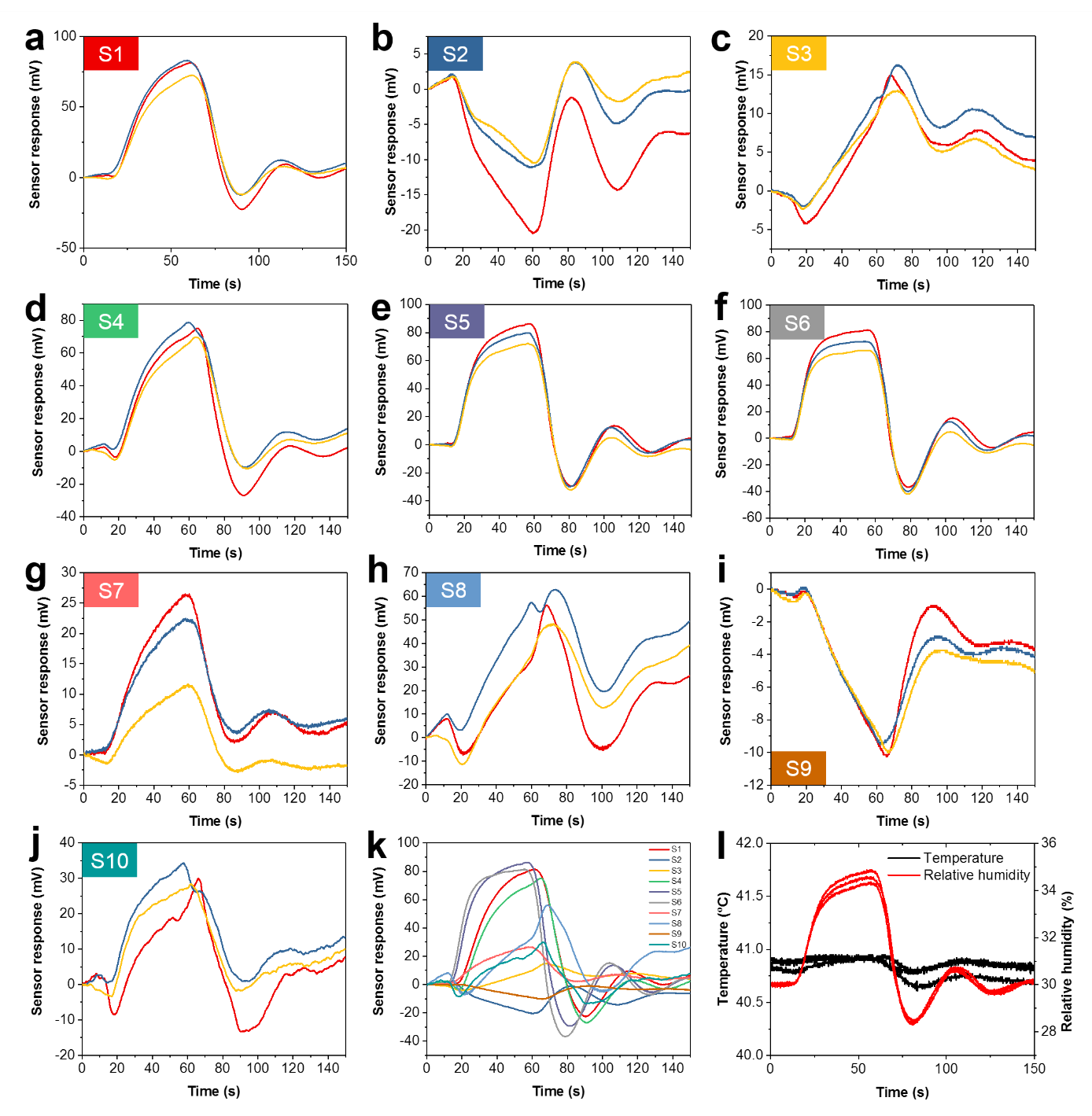


**Supplementary Figure 4.** **Cross-humidity test of GeNose C19. a-j** Responses of ten different gas sensors in GeNose C19 (S1 – S10) during cross-humidity tests. The humidity was modified in real time, while the temperature was kept at relative stable values. **k** Sensor array sensor signals during the first humidity test. **l** Temperature and humidity measured during cross-sensitivity assessment. From three repeated measurements, the sensors yielded similar responses indicating their reliable sensing results.

# Principal component analysis (PCA)


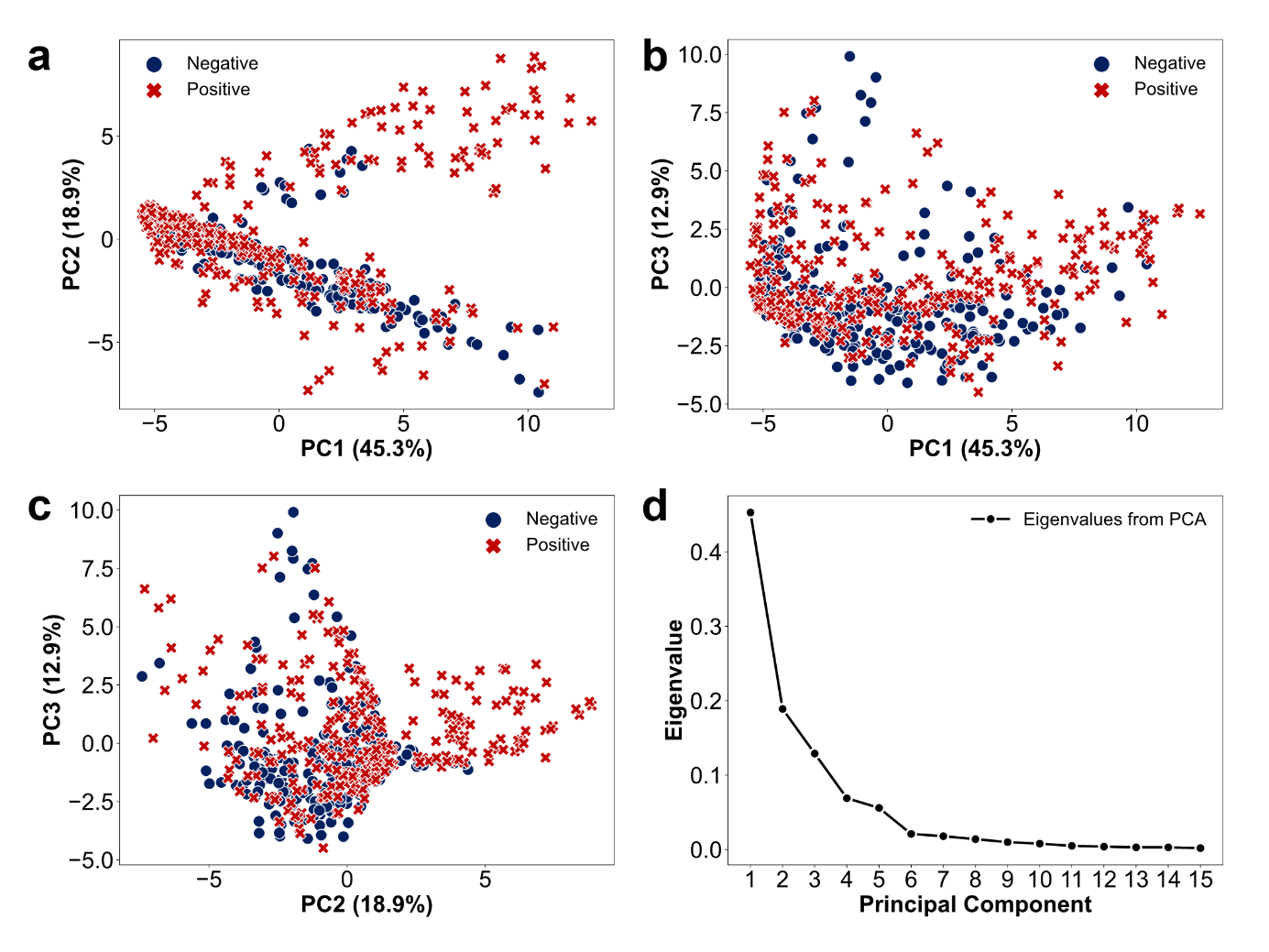


**Supplementary Figure 5.** **Principal component analysis (PCA) score plots of RT-qPCR-confirmed positive (red) and negative (blue) COVID-19.** **a** PC1 vs. PC2, **b** PC2 vs. PC3, **c** PC2 vs. PC3, and **d** eigenvalues of 15 components.

# Machine learning performance analysis


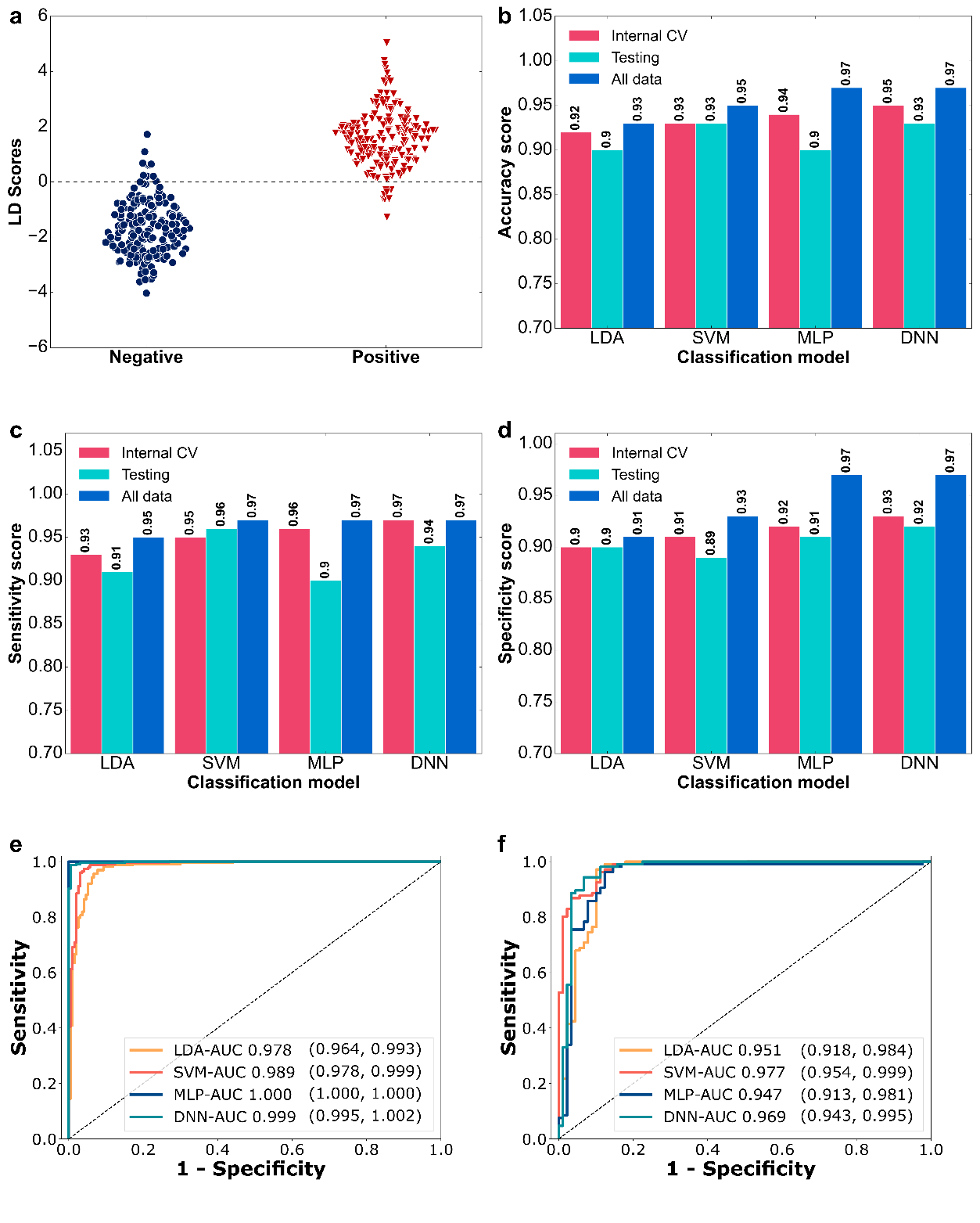


**Supplementary Figure 6.** **Machine learning performance analysis for the measured breath data based on subject group.** Eeach subject is considered as one breath sample. Subjects were categorized into positive and negative groups of COVID-19. In each group, they were randomly assigned for training and testing groups. All data were analyzed with a single initial breath sample from each subject (with total number of 83 subjects) based on the designated group. **a** Classification of two different exhaled breath samples (i.e., positive and negative COVID-19 patients) using the LDA model. **b** Overall accuracy (micro-averaged F1-score), **c** sensitivity, and **d** specificity of training (internal 10-fold cross-validation (CV)), testing, and all datasets obtained by four different machine learning algorithms (LDA, SVM, MLP, and DNN). **e and f** Receiver operating characteristic (ROC) curves of the training and testing data, respectively, using Area Under Curve (AUC) analysis, obtained by four different machine learning algorithms.

AUC confidence interval of ROC is defined using the normal distribution approach (sampling distribution approach). With a confidence interval of 1–α, the AUC confidence inverval is ± $se\cdot z_{crit}$. The value of *se* can be calculated employing Equation (S1), where *n_1_* and *n_2_* are the numbers of samples 1 and 2, respectively. The value of $z_{crit}$ can be calculated using the value of the z-score function of $1-\alpha/2$. In this study, the value of α is 0.05.

| $se=\sqrt{\frac{q_{0}+\left( n_{1}-1 \right)q_{1}+\left( n_{2}-1 \right)q_{2}}{n_{1}n_{2}}}$  $q_{0}=AUC\left( 1-AUC \right), q_{1}=\frac{AUC}{2-AUC}-AUC^{2}, q_{2}=\frac{2AUC^{2}}{1+AUC}-AUC^{2}$ | (S1) |
| --- | --- |

# Exhaled breath data processing steps


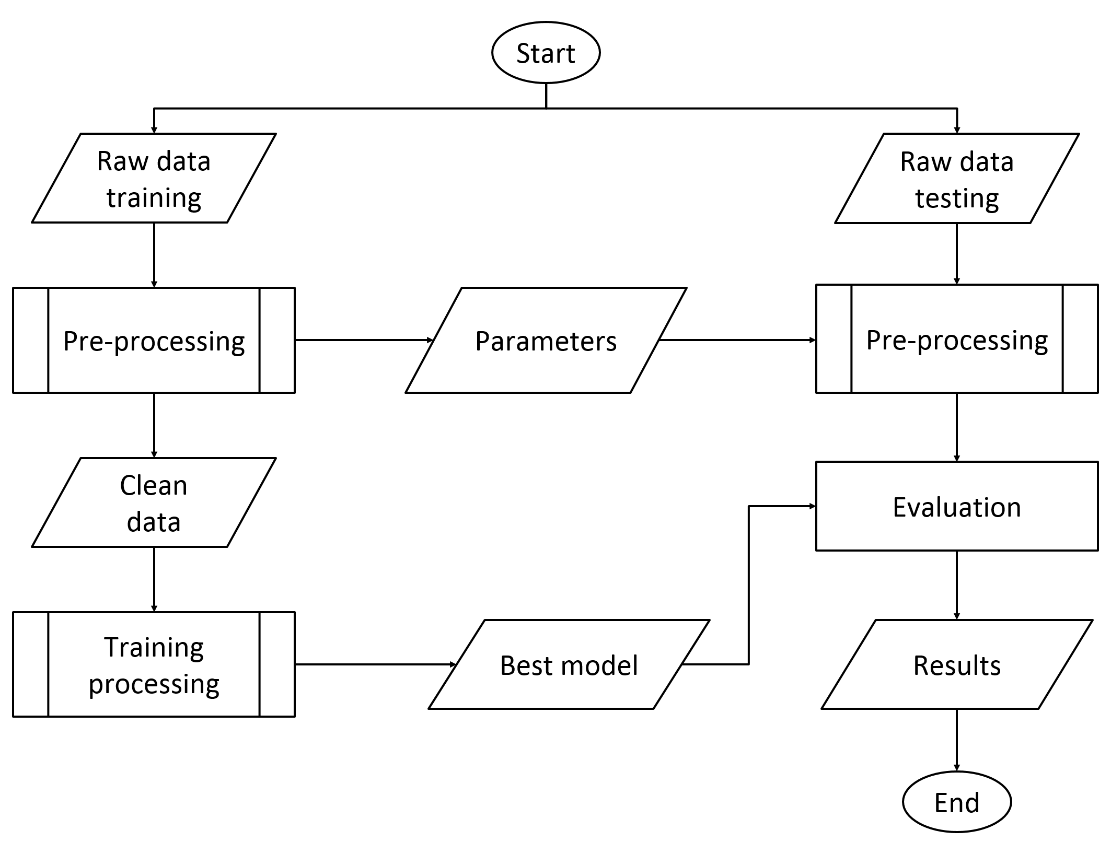


**Supplementary Figure 7.** **Block diagram of machine learning-based data processing steps used in GeNose C19 system.** Four different models (i.e., linear discriminant analysis (LDA), support vector machine (SVM), multilayer perceptron (MLP), and deep neural network (DNN)) were investigated to differentiate and classify the exhaled breath data.

# Non-parametric ANOVA test

**Supplementary Table 1.** **Non-parametric ANOVA test (Kruskal-Wallis test) of sensor responses towards exhaled breaths of positive and negative COVID-19 patients**. Four features (i.e., maximum, median, standard deviation, and variance) were extracted from responses of ten sensors (S1 – S10).

| **No.** | **Feature** | **Sensor** | **Positive** | | **Negative** | | **Statistic value** | ***p*-value** | **H0** |
| --- | --- | --- | --- | --- | --- | --- | --- | --- | --- |
|  |  |  | **Mean** | **Std** | **Mean** | **Std** |  |  |  |
| 1 | Maximum | **S1*** | 343.89 | 316.86 | 219.69 | 226.95 | 11.213 | 0.001 | Reject |
| 2 |  | S2 | 427.29 | 371.71 | 327.35 | 267.41 | 3.743 | 0.053 | Not-Reject |
| 3 |  | S3 | 146.14 | 204.64 | 142.49 | 145.08 | 2.874 | 0.09 | Not-Reject |
| 4 |  | S4 | 323.13 | 322.51 | 342.42 | 266.55 | 2.588 | 0.108 | Not-Reject |
| 5 |  | **S5*** | 304.14 | 300.57 | 402.78 | 289.35 | 21.32 | 0 | Reject |
| 6 |  | **S6*** | 129.91 | 156.77 | 72.23 | 93.91 | 20.304 | 0 | Reject |
| 7 |  | S7 | 265.48 | 248.51 | 224.64 | 220.64 | 1.341 | 0.247 | Not-Reject |
| 8 |  | S8 | 389.18 | 422.31 | 297.10 | 298.56 | 1.95 | 0.163 | Not-Reject |
| 9 |  | **S9*** | 517.61 | 473.33 | 205.67 | 241.83 | 41.745 | 0 | Reject |
| 10 |  | **S10*** | 215.68 | 332.79 | 53.34 | 70.28 | 35.381 | 0 | Reject |
| 11 | Median | **S1*** | 200.17 | 336.24 | 107.27 | 272.65 | 6.53 | 0.011 | Reject |
| 12 |  | S2 | 338.18 | 381.69 | 261.21 | 299.94 | 2.086 | 0.149 | Not-Reject |
| 13 |  | **S3*** | 38.24 | 195.17 | 56.38 | 194.44 | 8.202 | 0.004 | Reject |
| 14 |  | S4 | 243.63 | 267.12 | 261.70 | 226.76 | 2.494 | 0.114 | Not-Reject |
| 15 |  | **S5*** | 210.63 | 325.26 | 320.94 | 341.65 | 25.197 | 0 | Reject |
| 16 |  | S6 | 47.26 | 168.42 | 21.93 | 111.48 | 3.745 | 0.053 | Not-Reject |
| 17 |  | S7 | 143.54 | 250.47 | 107.76 | 287.07 | 0.225 | 0.635 | Not-Reject |
| 18 |  | S8 | 274.07 | 405.06 | 150.57 | 373.71 | 3.399 | 0.065 | Not-Reject |
| 19 |  | **S9*** | 367.83 | 480.54 | 106.08 | 286.46 | 23.252 | 0 | Reject |
| 20 |  | **S10*** | 147.77 | 293.67 | 24.54 | 77.82 | 15.207 | 0 | Reject |
| 21 | Standard deviation | **S1*** | 118.77 | 80.99 | 94.00 | 79.20 | 12.953 | 0 | Reject |
| 22 |  | S2 | 134.68 | 100.11 | 108.91 | 73.92 | 3.347 | 0.067 | Not-Reject |
| 23 |  | **S3*** | 47.46 | 50.34 | 51.87 | 50.46 | 7.259 | 0.007 | Reject |
| 24 |  | S4 | 93.36 | 84.73 | 107.23 | 86.28 | 2.88 | 0.09 | Not-Reject |
| 25 |  | **S5*** | 88.00 | 75.43 | 123.26 | 78.72 | 34.309 | 0 | Reject |
| 26 |  | **S6*** | 43.43 | 41.58 | 31.95 | 35.14 | 10.969 | 0.001 | Reject |
| 27 |  | S7 | 85.00 | 62.98 | 87.46 | 70.23 | 0.051 | 0.821 | Not-Reject |
| 28 |  | S8 | 111.67 | 101.97 | 114.84 | 81.48 | 3.68 | 0.055 | Not-Reject |
| 29 |  | **S9*** | 150.36 | 116.12 | 75.42 | 70.88 | 52.675 | 0 | Reject |
| 30 |  | **S10*** | 59.10 | 81.15 | 19.00 | 17.06 | 39.02 | 0 | Reject |
| 31 | Variance | **S1*** | 20665.89 | 26613.63 | 15108.07 | 28209.00 | 12.953 | 0 | Reject |
| 32 |  | S2 | 28160.64 | 34178.64 | 17325.57 | 21990.55 | 3.347 | 0.067 | Not-Reject |
| 33 |  | **S3*** | 4785.78 | 10405.56 | 5236.81 | 15701.41 | 7.259 | 0.007 | Reject |
| 34 |  | S4 | 15895.43 | 23890.76 | 18942.55 | 26418.83 | 2.88 | 0.09 | Not-Reject |
| 35 |  | **S5*** | 13433.46 | 23012.07 | 21389.87 | 25004.13 | 34.309 | 0 | Reject |
| 36 |  | **S6*** | 3615.11 | 8594.24 | 2255.90 | 9220.30 | 10.969 | 0.001 | Reject |
| 37 |  | S7 | 11190.39 | 15020.13 | 12581.33 | 22580.10 | 0.051 | 0.821 | Not-Reject |
| 38 |  | S8 | 22867.84 | 38770.38 | 19826.55 | 28451.64 | 3.68 | 0.055 | Not-Reject |
| 39 |  | **S9*** | 36092.04 | 46977.83 | 10712.47 | 23274.98 | 52.675 | 0 | Reject |
| 40 |  | **S10*** | 10077.74 | 30541.28 | 651.94 | 1479.80 | 39.02 | 0 | Reject |

# Pairwise difference between classification models

**Supplementary Table 2.** **Pairwise difference between classification models based on Cohen’s d equation**. Differentiation between algorithm and classification models is shown in terms of accuracy, sensitivity, and specificity. Large differentiation in performance is demonstrated in DNN compared to LDA, SVM and MLP.

| **Combination** | | **Accuracy** | | **Sensitivity** | | **Specificity** | |
| --- | --- | --- | --- | --- | --- | --- | --- |
|  |  | **Score** | **Interpretable** | **Score** | **Interpretable** | **Score** | **Interpretable** |
| LDA | SVM | 0.688 | Large | 0.598 | Medium | 0.482 | Medium |
| LDA | MLP | 0.828 | Large | 0.658 | Large | 0.362 | Medium |
| LDA | DNN | 1.693 | Very large | 1.709 | Very large | 0.661 | Large |
| SVM | MLP | 0.081 | Very small | 0.167 | Small | 0.096 | Very small |
| SVM | DNN | 2.888 | Very large | 4.137 | Very large | 1.663 | Very large |
| MLP | DNN | 3.459 | Very large | 3.251 | Very large | 1.342 | Very large |

# Gas chromatography-mass spectroscopy (GC-MS)

**Supplementary Table 3.** **Volatile organic compounds (VOCs) in the exhaled breaths of positive and negative COVID-19 patients measured by gas chromatography-mass spectroscopy (GC-MS).** Two sampled positive COVID-19 patients are indicated with N and S. Four investigated negative COVID-19 patients are written as R, K, NS, and SK.

|  | **Positive COVID-19 patients** | | | | | | **Negative COVID-19 patients** | | | | | | | | | | **Molecular formula** | **Volatile**  **category** | **Detecting sensor** |
| --- | --- | --- | --- | --- | --- | --- | --- | --- | --- | --- | --- | --- | --- | --- | --- | --- | --- | --- | --- |
| **Compounds** | **N1** | **N2** | **N3** | **S1** | **S2** | **S3** | **R1** | **R2** | **R3** | **K1** | **K2** | **K3** | **NS1** | **NS2** | **SK1** | **SK2** |  |  |  |
| Hydrogen cyanide |  |  | **√** | **√** | **√** | **√** | **√** | **√** | **√** | **√** | **√** | **√** | **√** | **√** | **√** | **√** | [HCN](https://pubchem.ncbi.nlm.nih.gov/#query=HCN) | Acid | S2, S6, S9 |
| Nitrogen | **√** | **√** | **√** | **√** | **√** | **√** | **√** | **√** | **√** | **√** | **√** | **√** | **√** | **√** | **√** | **√** | N_2_ |  |  |
| Carbon monoxide | **√** | **√** | **√** | **√** | **√** |  | **√** | **√** |  | **√** |  | **√** |  |  | **√** |  | CO |  | S1, S3, S4, S5, S6, S8 |
| Nickel tetracarbonyl | **√** |  |  | **√** |  | **√** |  |  | **√** |  |  | **√** |  | **√** | **√** | **√** | [C_4_NiO_4_](https://pubchem.ncbi.nlm.nih.gov/#query=C4NiO4) | Carbon | S1, S3, S4, S5, S5, S6, S8 |
| Butanedioic acid, 2-cyano-2,3-dimethyl-, diethyl ester |  | **√** |  |  |  |  | **√** |  |  |  |  |  |  |  |  |  | [C_11_H_17_NO_4_](https://pubchem.ncbi.nlm.nih.gov/#query=C11H17NO4) | Ester | S2, S9 |
| Manganese, acetylpentacarbonyl-, (OC-6-21)- | **√** |  |  |  |  |  |  |  |  | **√** |  |  |  |  |  |  | [C_7_H_3_M_n_O_6-_](https://pubchem.ncbi.nlm.nih.gov/#query=C7H3MnO6-) | Furan |  |
| Ethylene |  |  |  | **√** | **√** |  |  |  |  |  | **√** |  |  |  | **√** | **√** | [C_2_H_4_](https://pubchem.ncbi.nlm.nih.gov/#query=C2H4) | Hydrocarbon | S10 |
| Carbon monoxide | **√** | **√** | **√** | **√** | **√** | **√** | **√** | **√** | **√** | **√** | **√** | **√** | **√** | **√** | **√** | **√** | CO |  | S1, S3, S4, S5, S6, S8 |
| Nitrogen | **√** | **√** | **√** |  |  |  |  | **√** |  |  |  |  |  |  |  |  | N_2_ |  |  |
| Ethylene | **√** | **√** | **√** |  |  |  |  |  |  |  |  | **√** |  |  |  |  | [C_2_H_4_](https://pubchem.ncbi.nlm.nih.gov/#query=C2H4) | Hydrocarbon | S10 |
| Butanedioic acid, 2-cyano-2,3-dimethyl-, diethyl ester |  |  |  |  |  |  |  |  | **√** | **√** |  |  |  |  |  |  | [C_11_H_17_NO_4_](https://pubchem.ncbi.nlm.nih.gov/#query=C11H17NO4) | Ester | S2, S9 |
| Acetic acid, ethoxyhydroxy-, ethyl ester |  |  | **√** |  |  |  |  |  |  | **√** |  | **√** |  |  |  |  | [C_10_H_18_O_3_](https://pubchem.ncbi.nlm.nih.gov/#query=C10H18O3) | Ester | S2, S9 |
| Ethanol | **√** | **√** | **√** |  |  |  | **√** | **√** | **√** | **√** | **√** | **√** |  |  |  |  | [C_2_H_6_O](https://pubchem.ncbi.nlm.nih.gov/#query=C2H6O) | Alcohol | S1-S10 |
| Butyl aldoxime, 3-methyl-, syn- |  |  |  |  |  |  |  |  |  |  |  |  | **√** | **√** | **√** | **√** | [C_5_H_11_NO](https://pubchem.ncbi.nlm.nih.gov/#query=C5H11NO) | Hydrocarbon | S10 |
| Acetic acid, dimethoxy-, methyl ester |  |  |  |  |  |  |  |  |  |  | **√** |  |  |  |  |  | [C_5_H_10_O_4_](https://pubchem.ncbi.nlm.nih.gov/#query=C5H10O4) | Ester | S2, S9 |
| Cyclohexan-1,4,5-triol-3-one-1-carboxylic acid |  |  |  |  |  |  |  |  |  |  |  |  |  | **√** |  |  | [C_7_H_10_O_6_](https://pubchem.ncbi.nlm.nih.gov/#query=C7H10O6) | Acid | S2, S9 |
| Acetic acid, anhydride with formic acid |  |  |  |  |  |  |  |  |  |  |  |  |  |  | **√** |  | [C_10_H_18_O_3_](https://pubchem.ncbi.nlm.nih.gov/#query=C10H18O3) | Ester | S2, S9 |
| Pyridine, 2-nitro- |  |  |  |  |  |  |  |  |  |  |  |  |  | **√** |  |  | [C_5_H_4_N_2_O_2_](https://pubchem.ncbi.nlm.nih.gov/#query=C5H4N2O2) | Organic | S8,S9,S10 |
| Tetrahydropyrrole-3-ol-5-carboxylic acid, 1-acetyl-, methyl ester |  |  |  |  |  |  |  |  |  |  |  |  | **√** |  |  |  | [C_10_H_14_O_3_](https://pubchem.ncbi.nlm.nih.gov/#query=C10H14O3) | Ester | S2,S9 |
| Cis-3-Butyl-4-vinyl-cyclopentene |  |  |  |  |  |  |  |  |  |  |  |  |  | **√** |  |  | [C_11_H_18_](https://pubchem.ncbi.nlm.nih.gov/#query=C11H18) | Hydrocarbon | S10 |
| Bicyclo[2.2.1]heptane, 2,2-dimethyl-3-methylene-, (1S)- |  |  |  |  |  |  | **√** | **√** | **√** | **√** |  | **√** | **√** | **√** | **√** | **√** | [C_10_H_16_](https://pubchem.ncbi.nlm.nih.gov/#query=C10H16) | Hydrocarbon | S10 |
| 2,4-Heptadien-6-ynal, (E,E)- |  |  |  |  |  |  |  |  |  |  |  |  |  | **√** |  |  | [C_7_H_6_O](https://pubchem.ncbi.nlm.nih.gov/#query=C7H6O) | Hydrocarbon | S10 |
| Decane, 2,6,8-trimethyl- |  |  |  |  |  |  |  |  |  |  |  |  | **√** | **√** | **√** | **√** | [C_13_H_28_](https://pubchem.ncbi.nlm.nih.gov/#query=C13H28) | Hydrocarbon | S10 |
| Heptane, 2,2-dimethyl- |  |  |  |  |  |  | **√** |  |  |  |  |  |  |  |  |  | [C_10_H_16_](https://pubchem.ncbi.nlm.nih.gov/#query=C10H16) | Hydrocarbon | S10 |
| Oxalic acid, bis(isobutyl) ester |  |  |  |  |  |  |  |  | **√** |  |  |  |  |  |  |  | [C_10_H_18_O_4_](https://pubchem.ncbi.nlm.nih.gov/#query=C10H18O4) | Ester | S2,S9 |
| Cyclotetrasiloxane, octamethyl- |  |  |  |  |  |  |  |  |  | **√** |  |  |  |  |  |  | [C_8_H_24_O_4_Si_4_](https://pubchem.ncbi.nlm.nih.gov/#query=C8H24O4Si4) | Hydrocarbon | S10 |
| Benzene, [(1-methyl-2-propenyl)oxy]- |  |  |  |  |  |  |  |  |  |  |  |  | **√** | **√** | **√** | **√** | [C_14_H_18_O_2_](https://pubchem.ncbi.nlm.nih.gov/#query=C14H18O2) | Hydrocarbon | S10 |
| 1-Cyclopentene-1-propanoic acid, 2-[3,7-bis[(trimethylsilyl)oxy]-1-octenyl]-5-oxo-, methyl ester | **√** |  |  |  |  |  |  |  |  |  |  |  |  |  |  |  | [C_23_H_42_O_5_Si_2_](https://pubchem.ncbi.nlm.nih.gov/#query=C23H42O5Si2) | Ester | S2,S9 |
| Benzoic acid, hydrazide |  |  |  |  |  |  |  |  |  |  |  |  | **√** | **√** | **√** | **√** | [C_7_H_8_N_2_O](https://pubchem.ncbi.nlm.nih.gov/#query=C7H8N2O) | Ester | S2,S9 |
| 3-Hydroxymandelic acid, ethyl ester, di-TMS |  |  |  |  |  |  |  |  |  |  |  |  | **√** | **√** |  |  | [C_16_H_28_O_4_Si_2_](https://pubchem.ncbi.nlm.nih.gov/#query=C16H28O4Si2) | Ester | S2,S9 |
| 2,5-Dihydroxybenzaldehyde, 2TMS derivative | **√** | **√** | **√** | **√** | **√** | **√** | **√** | **√** | **√** | **√** | **√** | **√** |  |  |  |  | [C_13_H_22_O_3_Si_2_](https://pubchem.ncbi.nlm.nih.gov/#query=C13H22O3Si2) | Aldehyde | S2 |
| Isobornyl thiocyanoacetate | **√** |  |  |  |  |  |  |  |  |  |  |  |  |  | **√** |  | [C_13_H_19_NO_2_S](https://pubchem.ncbi.nlm.nih.gov/#query=C13H19NO2S) | Hydrocarbon | S10 |
| 1-Nonadecanol, TMS derivative | **√** | **√** | **√** | **√** | **√** | **√** | **√** | **√** | **√** | **√** | **√** | **√** |  |  |  |  | C_22_H_48_OSi | Alcohol | S1-S10 |
| 3,7,11,15-tetramethylhexadecan-1,2,3-triol silylated |  |  |  |  |  |  |  |  |  |  |  |  | **√** |  |  |  | [C_20_H_40_O](https://pubchem.ncbi.nlm.nih.gov/#query=C20H40O) | Alcohol | S1-S10 |
| Silane, dimethyl |  |  | **√** |  |  | **√** |  |  |  | **√** |  |  |  |  |  |  | [C_22_H_34_O_3_Si_2_](https://pubchem.ncbi.nlm.nih.gov/#query=C22H34O3Si2) | Hydrocarbon | S10 |
| Bicyclo[2.2.1]heptane, 2-chloro-2,3,3-trimethyl- |  |  |  |  |  |  |  |  |  |  |  |  |  | **√** |  |  | [C_10_H_17_Cl](https://pubchem.ncbi.nlm.nih.gov/#query=C10H17Cl) | Hydrocarbon | S10 |
| Cyclohexanol, 2-methyl-3-(1-methylethenyl)-, acetate, (1a,2a,3a)- |  |  |  |  |  |  |  |  |  |  |  |  |  | **√** | **√** |  | [C_12_H_20_O_2_](https://pubchem.ncbi.nlm.nih.gov/#query=C12H20O2) | Acid | S2,S9 |
| 3-Isopropoxy-1,1,1,7,7,7-hexamethyl-3,5,5-tris(trimethylsiloxy)tetrasiloxane | **√** | **√** | **√** | **√** | **√** | **√** | **√** | **√** | **√** | **√** | **√** | **√** | **√** | **√** | **√** | **√** | [C_18_H_52_O_7_Si_7_](https://pubchem.ncbi.nlm.nih.gov/#query=C18H52O7Si7) | Hydrocarbon | S10 |
| Molybdenum, tricarbonyl-?-6-spiro[1,3-dioxolane-2,1'-benzocyclobutene) | **√** |  |  |  |  |  |  |  |  |  |  |  |  |  |  |  | [C_17_H_28_O_2_](https://pubchem.ncbi.nlm.nih.gov/#query=C17H28O2) | Hydrocarbon | S10 |
| 3-Butoxy-1,1,1,7,7,7-hexamethyl-3,5,5-tris(trimethylsiloxy)tetrasiloxane |  |  |  |  |  |  | **√** | **√** | **√** | **√** | **√** | **√** |  |  | **√** | **√** | [C_17_H_50_O_7_Si_7_](https://pubchem.ncbi.nlm.nih.gov/#query=C17H50O7Si7) | Hydrocarbon | S10 |
| 1-[2,4-Bis(trimethylsiloxy)phenyl]-2-[(4-trimethylsiloxy)phenyl]propan-1-one |  |  |  |  |  |  |  |  |  | **√** | **√** | **√** |  |  |  |  | [C_24_H_38_O_4_Si_3_](https://pubchem.ncbi.nlm.nih.gov/#query=C24H38O4Si3) | Hydrocarbon | S10 |
| Phenol, 2-benzyloxy-3,6-difluoro- |  |  |  |  |  |  | **√** |  |  | **√** |  |  |  |  |  |  | [C_13_H_12_O_2_](https://pubchem.ncbi.nlm.nih.gov/#query=C13H12O2) | Alcohol | S1-S10 |
