# Supplementary material for: Fast and noninvasive electronic nose for sniffing out COVID-19 based on exhaled breath-print recognition
Source: NPJ Digit Med. 2022 Aug 16;5:115. doi: 10.1038/s41746-022-00661-2 (PMC9379872; doi:10.1038/s41746-022-00661-2)
Supplement: Supplementary file 2 — COE English Editing [file 41746_2022_661_MOESM2_ESM.pdf]

enago www.enago.com | voxtab www.voxtab.com | ulatus www.ulatus.com

CERTIFICATE OF EDITING

This is to certify that the paper titled **Fast and noninvasive electronic nose for sniffing out COVID-19 based on exhaled breath-print recognition** commissioned to us has been edited for English language and spelling by Enago, an editing brand of Crimson Interactive Inc.

bsi. ISO/IEC 27001 Information Security Management
ISO/IEC 27001:2013 Certified

bsi. ISO 9001 Quality Management
ISO 9001:2015 Certified

Issued by:
Enago, Crimson Interactive Inc.
1732, 1st Ave #22627
New York, 10128
Phone: +1-877-712-2177

CRIMSON INTERACTIVE INC. U.S.A.

Disclaimer: The author is free to accept or reject our changes in the document after our editing. However, we do not bear responsibility for revisions made to the document after our edit on **July 5th, 2022.**

Global	www.enago.com, www.voxtab.com, www.ulatus.com
Japan	www.enago.jp, www.voxtab.jp, www.ulatus.jp
Brazil	www.enago.com.br
Germany	www.enago.de
Turkey	www.enago.com.tr
China	www.enago.cn
Taiwan	www.enago.tw

About Crimson:
Crimson Interactive Inc. provides English language editing, transcription, and translation services to individuals and corporate customers worldwide.
