# Supplementary material for: Fast and noninvasive electronic nose for sniffing out COVID-19 based on exhaled breath-print recognition
Source: NPJ Digit Med. 2022 Aug 16;5:115. doi: 10.1038/s41746-022-00661-2 (PMC9379872; doi:10.1038/s41746-022-00661-2)
Supplement: Supplementary file 3 — Ethical Clearance Document [file 41746_2022_661_MOESM3_ESM.pdf]

MEDICAL AND HEALTH RESEARCH ETHICS COMMITTEE (MHREC)
FACULTY OF MEDICINE, PUBLIC HEALTH AND NURSING
UNIVERSITAS GADJAH MADA – DR. SARDJITO GENERAL HOSPITAL

ETHICS COMMITTEE APPROVAL

Ref. No. : KE/FK/0665/EC/2020

Title of the Research Protocol	:	Pengembangan Prototip Electronic Nose Sebagai Alat Diagnosis Cepat Infeksi SARS-CoV2 Melalui Hembusan Nafas Pasien Rawat Inap Isolasi di RS Bhayangkara
Document(s) Approved and version	:	1. Study Protocol version 03 2020<br>2. Information for Subjects version 02 2020<br>3. Informed consent form version 02 2020
Principle Investigator	:	Dr. Eng. Kuwat Triyana, M.Si.
Participating Investigator(s)	:	1. dr. Dian Kesumapramudya Nurputra, M.Sc., Ph.D., Sp.A.<br>2. Dr. Ahmad Kusumaatmaja<br>3. dr. M. Hakim, M.Sc., Ph.D.
Date of Approval	:	15 JUN 2020<br>(Valid for one year beginning from the date of approval)
Institution(s)/place(s) of research	:	1. Lab Fisika Material dan Instrumentasi FMIPA UGM<br>2. Rawat Inap Isolasi di RS Bhayangkara<br>3. Lab Mikrobiologi FKKMK

The Medical and Health Research Ethics Committee (MHREC) states that the document above meets the ethical principle outlined in the International and National Guidelines on ethical standards and procedures for researches with human beings.

The Medical and Health Research Ethics Committee (MHREC) has the right to monitor the research activities at any time.

The investigator(s) is/are obliged to submit:

- ☑ Progress report as a continuing review (state its due time)
- ☑ Report of any serious adverse events (SAE)
- ☑ Final report upon the completion of the study

Prof. dr. Tri Wibawa, Ph.D., Sp.MK(K).
Panel's chairperson

dr. Yana Supriatna, Sp.Rad(K)., Ph.D
Panel's secretary

P.S: This letter uses signature scan of the panel's chairperson and Secretary of the Ethics Committee. The hardcopy official letter with authority's signature will be issued when it is possible and are kept as an archive of the Ethics Committee	Validation number : 5ee982f739d92 (http://komisietik.fk.ugm.ac.id/validasi)
