# Supplementary material for: Fast and noninvasive electronic nose for sniffing out COVID-19 based on exhaled breath-print recognition
Source: NPJ Digit Med. 2022 Aug 16;5:115. doi: 10.1038/s41746-022-00661-2 (PMC9379872; doi:10.1038/s41746-022-00661-2)
Supplement: Supplementary file 4 — clinical trial approval letter [file 41746_2022_661_MOESM4_ESM.pdf]

KEMENTERIAN KESEHATAN REPUBLIK INDONESIA
DIREKTORAT JENDERAL KEFARMASIAN DAN ALAT KESEHATAN
Jalan H.R. Rasuna Said Blok X-5 Kavling 4 - 9 Jakarta 12950
Telepon : (021) 5201590 Pesawat 2029, 8011
Faksimile : (021) 5296-4838 Kotak Pos : 203

GERMAS

Nomor	: FR.01.04/V/1550/2020	Jakarta, 2 November 2020
Lampiran	: -	
Hal	: Persetujuan Pelaksanaan Uji Klinik Prapemasaran	

Yang Terhormat,
Pimpinan PT. Swayasa Prakarsa
Di
Tempat

Menanggapi surat permohonan uji klinik dari PT. Swayasa Prakarsa tanggal 16 Oktober 2020 yang kami terima pada tanggal 28 Oktober 2020 dengan rincian:

Judul Protokol Uji Klinik	:	Uji Diagnostik GENOSVID untuk Deteksi Dini COVID-19: Inovasi Electronic-Nose dari UGM untuk Indonesia
Nama Peneliti utama	:	dr. Dian K. Nurputra, M.Sc, Ph.D, SpA
Pemohon	:	PT. Swayasa Prakarsa

maka setelah dievaluasi dapat diberikan PERSETUJUAN.

Persetujuan ini berlaku 2 (dua) tahun sejak tanggal ditetapkan. Selama masa berlaku persetujuan ini Saudara <u>diwajibkan</u> untuk:

1. melaporkan perkembangan pelaksanaan Uji Klinik yang dilakukan kepada Direktur Jenderal:
   a. selambatnya setiap 6 (enam) bulan;
   b. saat berakhirnya pelaksanaan Uji Klinik; dan/atau

c. jika terjadi penghentian pelaksanaan Uji Klinik sebelum waktunya dengan menjelaskan alasannya
2. bersedia sewaktu-waktu dilakukan Inspeksi CUKAKB terhadap tempat pelaksanaan Uji Klinik dan/atau tempat lain yang terkait dengan pelaksanaan Uji Klinik
3. melaporkan setiap Kejadian Tidak Diinginkan yang Serius (KTDS) yang terjadi di tempat uji dan KTDS dari Uji Klinik di negara lain yang melibatkan Tempat Pelaksanaan Uji Klinik di Indonesia
4. menyerahkan dokumen Uji Klinik yang mengalami perubahan

Jakarta, 2 November 2020
Direktur Jenderal,

KEMENTERIAN KESEHATAN REPUBLIK INDONESIA
DIREKTUR JENDERAL KEFARMASIAN DAN ALAT KESEHATAN

Dra. Engko Sosialine Magdalene, Apt., M.Bio Med
NIP. 19610119 198803 2 001

Tembusan:
1. Direktur Jenderal Pelayanan Kesehatan
2. Ketua Tim Evaluasi Dokumen Uji Klinik Alat Kesehatan, Prof. Dr. Akmal Taher, Sp.U(K).
